# Characterizing the Diverse Mutational Pathways Associated with R5-Tropic Maraviroc Resistance: HIV-1 That Uses the Drug-Bound CCR5 Coreceptor

**DOI:** 10.1128/JVI.01384-15

**Published:** 2015-09-02

**Authors:** Xiaowei Jiang, Felix Feyertag, Conor J. Meehan, Grace P. McCormack, Simon A. Travers, Charles Craig, Mike Westby, Marilyn Lewis, David L. Robertson

**Affiliations:** a Computational and Evolutionary Biology, Faculty of Life Sciences, University of Manchester, Manchester, United Kingdom; b Wellcome Trust Centre for Human Genetics, University of Oxford, Oxford, United Kingdom; c Department of Biology, University of Nevada, Reno, Nevada, USA; d School of Natural Sciences, National University of Ireland Galway, Galway, Ireland; e Department of Biomedical Sciences, Institute of Tropical Medicine, Antwerp, Belgium; f South African National Bioinformatics Institute, South African MRC Bioinformatics Unit, University of the Western Cape, Bellville, South Africa; g The Research Network, Sandwich, United Kingdom; h Pfizer R&D, Sandwich, United Kingdom

## Abstract

Entry inhibitors represent a potent class of antiretroviral drugs that target a host cell protein, CCR5, an HIV-1 entry coreceptor, and not viral protein. Lack of sensitivity can occur due to preexisting virus that uses the CXCR4 coreceptor, while true resistance occurs through viral adaptation to use a drug-bound CCR5 coreceptor. To understand this R5 resistance pathway, we analyzed >500 envelope protein sequences and phenotypes from viruses of 20 patients from the clinical trials MOTIVATE 1 and 2, in which treatment-experienced patients received maraviroc plus optimized background therapy. The resistant viral population was phylogenetically distinct and associated with a genetic bottleneck in each patient, consistent with *de novo* emergence of resistance. Recombination analysis showed that the C2-V3-C3 region tends to genotypically correspond to the recombinant's phenotype, indicating its primary importance in conferring resistance. Between patients, there was a notable lack of commonality in the specific sites conferring resistance, confirming the unusual nature of R5-tropic resistance. We used coevolutionary and positive-selection analyses to characterize the genotypic determinants of resistance and found that (i) there are complicated covariation networks, indicating frequent coevolutionary/compensatory changes in the context of protein structure; (ii) covarying sites under positive selection are enriched in resistant viruses; (iii) CD4 binding sites form part of a unique covariation network independent of the V3 loop; and (iv) the covariation network formed between the V3 loop and other regions of gp120 and gp41 intersects sites involved in glycosylation and protein secretion. These results demonstrate that while envelope sequence mutations are the key to conferring maraviroc resistance, the specific changes involved are context dependent and thus inherently unpredictable.

**IMPORTANCE** The entry inhibitor drug maraviroc makes the cell coreceptor CCR5 unavailable for use by HIV-1 and is now used in combination antiretroviral therapy. Treatment failure with drug-resistant virus is particularly interesting because it tends to be rare, with lack of sensitivity usually associated with the presence of CXCR4-using virus (CXCR4 is the main alternative coreceptor HIV-1 uses, in addition to CD4). We analyzed envelope sequences from HIV-1, obtained from 20 patients who enrolled in maraviroc clinical trials and experienced treatment failure, without detection of CXCR4-using virus. Evolutionary analysis was employed to identify molecular changes that confer maraviroc resistance. We found that in these individuals, resistant viruses form a distinct population that evolved once and was successful as a result of drug pressure. Further evolutionary analysis placed the complex network of interdependent mutational changes into functional groups that help explain the impediments to the emergence of maraviroc-associated R5 drug resistance.

## INTRODUCTION

Human immunodeficiency virus type 1 (HIV-1), the causative agent of human AIDS, uses two main immune cell surface proteins, CD4 and a chemokine coreceptor, to enter cells. Pairs of viral envelope proteins (the heavily asparagine [N]-glycosylated gp120-gp41 heterodimers) form trimeric complexes anchored on the virus surface through gp41, and these facilitate entry into cells (1). CD4 receptor binding initiates virus-cell membrane interaction, and the coreceptors CCR5 (C-C chemokine receptor type 5) and CXCR4 (C-X-C chemokine receptor type 4) further facilitate viral entry into cells. HIV-1 that exclusively uses CCR5 is termed R5 tropic, whereas HIV-1 that exclusively uses CXCR4 is termed X4 tropic; viruses that use both coreceptors are termed dual or mixed tropic (R5X4) (2). Members of the CCR5 antagonist drug class, also known as HIV-1 entry inhibitors, such as maraviroc, are negative allosteric modulators and prevent HIV-1 from entering the host cell (3).

For most drugs, a defined set of point mutations in viral genes are associated with resistance to a specific drug (4). Lack of sensitivity to maraviroc can arise in two ways. In the first mode, suppression of maraviroc-sensitive R5 viruses revealed preexisting CXCR4-using variants (5, 6). As maraviroc has no direct impact on strains using the CXCR4 coreceptor, patients are routinely screened for the presence of CXCR4-using viruses prior to treatment (tropism testing). The aim is to distinguish a viral population that is R5 from a viral population harboring either X4 or R5X4 variants. In the second mode, true resistance arises, where R5-tropic viruses adapt to use the maraviroc-bound CCR5 coreceptor (7, 8). Previous studies have documented that R5-tropic resistance to maraviroc is associated with point mutations in variable region 3 (the V3 loop) of gp120 (9, 10). However, no shared (and therefore predictable) set of sites of amino acid changes that conferred resistance in different patients have been reported. In addition, recent evidence indicated that R5 populations, as a consequence of mutations outside the V3 loop either in gp120 or in gp41, can also develop resistance (11, 12). Given the structural heterogeneity and the highly N-glycosylated nature of HIV-1's envelope, such that specific compensatory/coevolutionary changes are required to maintain protein stability and full functionality under drug selective pressure (13–18), it is not surprising that maraviroc-associated resistance is more diverse than that to other drug classes.

In this study, we investigated the emergence of resistance to maraviroc associated with use of the drug-bound CCR5 receptor by R5-tropic viruses. We analyzed full envelope sequences cloned from viruses obtained from 20 patients (17 maraviroc treated and 3 treated with placebo) enrolled in the MOTIVATE 1 and 2 clinical trials (19), a sample of those who failed without evidence of a CXCR4-using virus population out of a total of 1,049 patients. Experimental phenotypic characterization using the Monogram PhenoSense Entry assay was performed on these sequences to establish whether they were associated with a sensitive or resistant phenotype. This determined whether they were or were not able to enter the cell when maraviroc was bound to the CCR5 receptor, i.e., were susceptible or resistant, respectively. Analyzing these data, we confirmed the unusual nature of R5-tropic maraviroc resistance, and by performing evolutionary and structural analyses, we found complicated coevolutionary dynamics in maraviroc-sensitive and -resistant viruses. Specifically, we observed that there were strong coevolutionary changes between sites involved in CD4 binding, V3, sites in the potential N-linked glycosylation motifs (PNGMs) (N-X-S/T-X, where X represents any amino acid except proline [20]), and the signal peptide. These changes were associated with the important physicochemical properties of the covarying amino acids: hydrophobicity, molecular weight, and structure and/or function of the envelope protein. Collectively, our results explain the unpredictable nature of the evolutionary changes associated with the emergence of resistance to maraviroc in HIV-1's envelope protein.

## MATERIALS AND METHODS

### Data.

In the clinical studies MOTIVATE 1 and 2, 1,049 patients were enrolled and screened for R5-tropic virus. They were treatment-experienced patients with an HIV-1 RNA level of at least 5,000 copies/ml at screening who had received three classes of drugs and/or were infected with virus resistant to two drug classes. A number of these patients subsequently experienced therapy failure with an R5-tropic viral population. Virus from these patients was assessed using the PhenoSense Entry Assay. The HIV-1 envelope coding sequence was amplified from virus samples by PCR and ligated into a pCXAS expression vector to create an envelope expression vector library. Virus particles were produced by transfecting HEK293 cells with the purified envelope expression vector library and an HIV-1 genomic vector lacking the envelope-encoding region and containing a firefly luciferase gene. The ability of the pseudoviruses to infect U87 CD4^+^ cells overexpressing CCR5 in the presence or absence of various concentrations of inhibitor was assessed by measuring luciferase-generated relative light units (RLU). If increasing concentrations of maraviroc failed to give 100% inhibition and a plateau was observed, then the maximal percentages of inhibition (MPI) were estimated visually from the inhibition curve (7). MPI values of less than 95% were considered resistant (7). Resistant samples were further analyzed by clonal analysis. One hundred to 200 individual *env* clones from each sample were prescreened for viability and tropism in single-well cultures of CCR5- or CXCR4-expressing cells. A number of viable clones from 20 patients were selected for further phenotypic and genotypic analysis.

Full-length clonal envelope sequences were obtained before treatment and at one or more on-treatment time points from patients with viral loads of >500 copies/ml. An average of 15 sensitive and 12 resistant clonal sequences were obtained per patient. In addition to optimized background treatment, 17 patients (1 to 17) were given maraviroc, and 3 patients (18 to 20) were from the placebo-treated arm. All samples were confirmed R5 tropic using the original Monogram Trofile assay. RLU values for CCR5 entry efficiency were retrieved from the assay for each clone.

An initial examination of the relationship between gp160 sequence identity and RLU scores, regardless of drug susceptibility, revealed that identical sequences varied in their CCR5 RLU values by up to 500,000 RLU. In addition, V3 loops that were identical between clones were associated with RLU values that differed by up to 3 million RLU. This suggests that the individual amino acid sequence of the V3 loop or the full gp160 does not have a direct correlation with the efficiency value for CCR5 usage (represented by the RLU). Despite this discrepancy, all the sequences in this study represent clones from CCR5-using viruses (see Fig. S1 in the supplemental material).

The MPI score was not directly related to the individual amino acid sequence of either the full gp160 or the V3 loop, with identical sequences exhibiting different MPI scores (data not shown). To account for potential conflicts in sequence analysis resulting from these ambiguities, we used the pooled MPI scores (calculated by averaging the MPI scores for all the clones sampled from a patient) to group sensitive and resistant sequences, as they clustered together in all cases in the phylogenetic analysis. Due to a lack of scores of pooled resistant MPI, patient 17 was excluded from the coevolutionary analysis, because an imbalance of sequence numbers might hamper this type of analysis when comparing the results between sensitive and resistant viruses (21). For the between-patient analysis, this resulted in 182 and 181 sequences for sensitive and resistant viruses, respectively, in the group of patients receiving maraviroc, while in the placebo arm, it resulted in 34 sequences for the two time points (before and after treatment).

### Sequence alignment.

Amino acid sequences were aligned using MUSCLE (22), and this alignment was used as a template for aligning nucleotide sequences with respect to the reading frame. Alignments were checked manually using Jalview (23), and Jalview was used to calculate the consensus sequences for between-patient and within-patient alignments (23). Due to alignment ambiguity, portions of the gp160 sequence alignments were removed. These positions corresponded to (HXB2 amino acid numbering) 132 to 149, 151 to 153, 186 to 190, 352 to 355, 396 to 413, and 460 to 465. Sequence alignments are available upon request.

### Recombination analysis.

Recombination plays an important role in HIV evolution, which can also lead to obscure results in phylogenetic and downstream analyses (24). To account for this, we performed recombination analysis using Recco software, which employs an implementation of a dynamic programming algorithm for detecting likely parental sequences and breakpoints from within an alignment by finding a path that minimizes costs based on mutations and putative recombination (25). For each patient, sequences were pooled, and 1,000-replicate permutations were performed to assess the reliability of recombination between individual strains within a patient. This provides *P* value support for recombination for an alignment, as well as for individual breakpoints. The nucleotide sequence alignment for each patient was analyzed individually, and sequences that were identified as being likely recombinants (*P* < 0.05), were further investigated. Of particular interest were recombinant sequences that had resistant and sensitive parental sequences (see Results).

These recombinant sequences involving sensitive and resistant viruses were removed from the phylogenetic and positive-selection analyses. A resistant virus from patient 10 (sequence number 18) that clustered with sensitive viruses in the initial phylogenetic analysis (not shown) was analyzed with RDP (26) and found to exhibit evidence of recombination and so was also removed.

### Molecular phylogenetic analysis.

BEAST v1.8.1 was used to construct dated trees and to infer the variance of the effective population size over time using Bayesian skyline plots (27, 28). Separate trees were constructed for each patient from nucleotide sequence alignments (excluding the recombinant sequences detected as described above), using an HKY model of nucleotide substitution with gamma-distributed rate heterogeneity, a proportion of invariant sites, an uncorrelated lognormal relaxed molecular clock, and a Bayesian skyline coalescent tree prior with tip heights set to the number of days since the trial began. BEAST XML files were created using BEAUTi v1.8.1, and chains were run for 10^8^ states and inspected using Tracer v1.6. Stepwise maximum clade credibility phylogenetic trees were created from the BEAST MCMC tree log file with a burn-in of 10% using TreeAnnotator v1.8.1, and stepwise Bayesian skyline plots were generated using Tracer v1.6. The BEAST XML files are available upon request.

### Coevolutionary analysis.

To understand the nonindependent evolution of amino acid sites in the envelope protein with and without drug selection, we used the coevolutionary method, CAPS, to detect sites undergoing coevolutionary changes using protein sequences (29–31). This method uses a strategy to identify structurally and/or functionally important covarying sites (inferred to be coevolving) that can be explained by compensatory physicochemical changes (with negative or positive correlations) (29–31). First, the method normalizes sequences by Poisson distance in the alignment to minimize the effects of phylogenetic coevolution. Second, it identifies statistically significant pairs of covarying sites by comparing a calculated BLOSUM62 score correlation coefficient to a randomly generated distribution (30, 31), where we use a high correlation coefficient cutoff (ρ ≥ 0.9) for initial filtering, a small alpha value (0.001), and 1 million random samples for the statistical test to minimize false positives. Third, it continues to look for covarying pairs by two important physicochemical properties in the results: hydrophobicity and molecular weight. The degree of connectivity in the resulting networks was measured by calculating the connection coefficient as 2*e*/(*n* × [*n* − 1]), where *e* and *n* correspond to the number of edges and nodes in the network, respectively. Finally, we visualized and analyzed the covariation networks with all the information associated with nodes and edges in Cytoscape (version 2.8.3) (32, 33). The reference HIV-1 variant HXB2 was used for domain mapping (http://www.uniprot.org/uniprot/P04578). DOG (version 2.0) was used to illustrate HXB2 domain structures for covarying sites in the figures (34).

### Prediction of potential N-linked glycosylation sites.

The NetNGlyc 1.0 server was used to predict potential N-linked glycosylation sites and motifs (http://www.cbs.dtu.dk/services/NetNGlyc/). The default cutoff value 0.5 was used for considering potential N-linked glycosylation sites/motifs (N-X-S/T-X). In the between-patient coevolutionary analysis, consensus sequences of sensitive and resistant viruses were used for the prediction. In the within-patient analysis, consensus sequences from individual patients were used for the prediction. To understand the significance of observed glycosylation sites in covariation networks, we used χ^2^ tests with the following equations:

(1) $$f_{e}=\frac{n}{N}$$

(2) $$E_{i}=f_{e}N_{i}$$

(3) $$X^{2}=\frac{\left(O_{i}-E_{i}\right)}{E_{i}}$$

In equation 1, *f_e_* represents the expected frequency of the number of N-linked glycosylation sites/motifs, *n* denotes the predicted number of N-linked glycosylation sites/motifs in the envelope proteins, and *N* denotes the total number of sites in the alignment/consensus sequence. In equation 2, *E_i_* represents the expected number of N-linked glycosylation sites/motifs in a given number of covarying sites (*N_i_*), and *O_i_* denotes the observed number of N-linked glycosylation sites/motifs predicted in a given covariation network. For a given network *i*, the χ^2^ test was performed with equation 3 and 1 degree of freedom (at a 95% significance level). Due to the small number of observed glycosylation sites present in some cases, we also used Fisher exact tests.

### Positive-selection analysis.

We used HyPhy to detect positively selected sites (with posterior probability *P* > 0.95) in sensitive and resistant viruses by comparing four models: M1 (neutral), M2 (selection), M7 (beta), and M8 (beta and ω) (35, 36). HyPhy used the aligned codon sequences and the corresponding maximum-likelihood (ML) phylogenetic tree. RAxML MPI was used to construct the ML tree with 100 bootstraps (37). We used ProtTest (with PhyML as its key component) to select the best-fit amino acid substitution model for the alignment (38, 39).

### Nucleotide sequence accession numbers.

Sequences were deposited in GenBank under accession numbers KT452084 to KT452622.

## RESULTS

### Inference of evolutionary history and recombination analysis.

**FIG 1:**
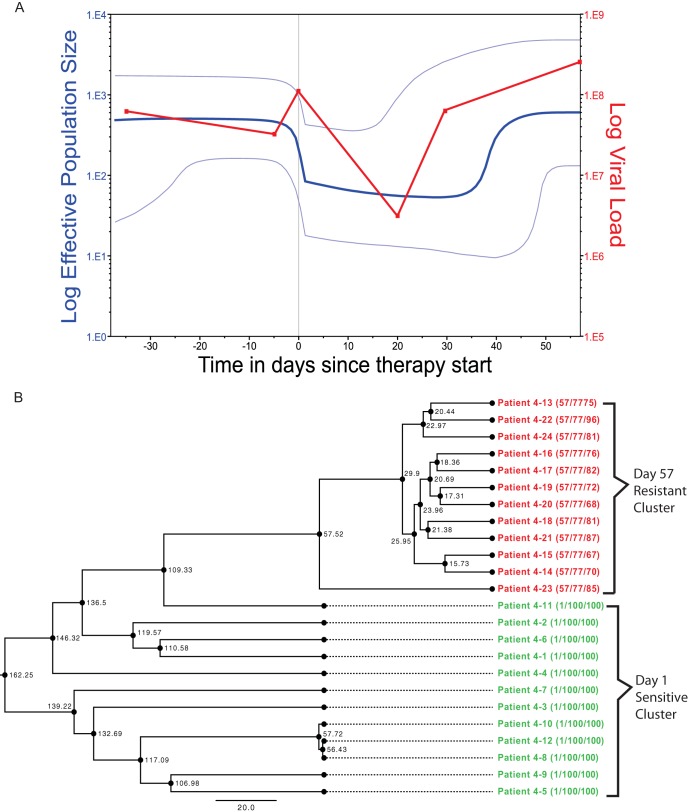
Evolutionary analysis. Patient 4 is presented as a representative example. Phylogenetic trees and Bayesian skyline plots for patients 1 to 16 and placebo arm patients 18 to 20 are presented in Fig. S2A, B, and C in the supplemental material. (A) Bayesian skyline plot showing a sharp drop (blue line) in genetic diversity as therapy starts, with a rebound occurring as drug resistance develops, which is consistent with the viral-load plot of the patient (red line). The light-blue lines represent the 95% highest posterior density (HPD) confidence intervals. (B) Phylogenetic tree for patient 4 created with BEAST using a Bayesian skyline coalescent. The tree tips are calibrated by days into therapy, with the R5-sensitive strains sampled at day 1 in green and the R5-resistant strains sampled at day 57 in red. Patient and sequence numbers are shown at the tip nodes, as well as sampling day and pooled MPI, with the sequence MPI shown in parentheses. The internal nodes show the ancestral time points in days.

We inferred evolutionary relationships from the clonal sequence data sampled before and after treatment (depicted for patient 4 in Fig. 1; see Fig. S2A and B in the supplemental material for all patients' viral phylogenies). In all 17 maraviroc-treated patients, the overwhelming majority of resistant viruses (second and subsequent time points) form a monophyletic cluster emerging from the sensitive population (the first time point). Acquisition of R5-tropic maraviroc resistance is thus clearly associated with the emergence of resistant variants from the existing susceptible population as opposed to the presence of preexisting (divergent) variants, as is the case with the emergence of CXCR4-using viruses (5, 6). This *de novo* emergence of resistant virus was confirmed by Bayesian skyline analysis, which detected a distinct drop, by up to a factor of 10, in the genetic diversity, indicating a bottleneck in the size of the viral population between the time points, confirmed by reduced HIV-1 RNA concentrations (Fig. 1 shows an example; see Fig. S2A and B in the supplemental material for all maraviroc-treated patients' analyses). Interestingly, a rebound in the genetic diversity to preresistance levels (again confirmed by viral loads) was observed in patients 1, 3, 4, 5, 6, 8, 9, 10, 11, and 13 after resistance developed (see Fig. S2A and B in the supplemental material). In the three patients from the placebo arm of the MOTIVATE trials, we found no significant shift of viral genetic diversity between time points, except for patient 20, who had a very low CD4 cell count at the starting time point (see Fig. S2C in the supplemental material). As no maraviroc was present, this drop in genetic diversity in patient 20 was presumably associated with the efficacy of the background treatment. Collectively, this indicates that the resistance to CCR5 antagonist maraviroc in the R5 viral population evolves *de novo*, and unlike CXCR4-using virus, the associated resistance is not due to an already present minority population of resistant virus that becomes dominant under drug selective pressure. Note that, while we cannot discount a resistant variant being present by chance prior to treatment, our results are consistent, unlike CXCR4-associated resistance, with no preexisting resistant population of any significance.

**FIG 2:**
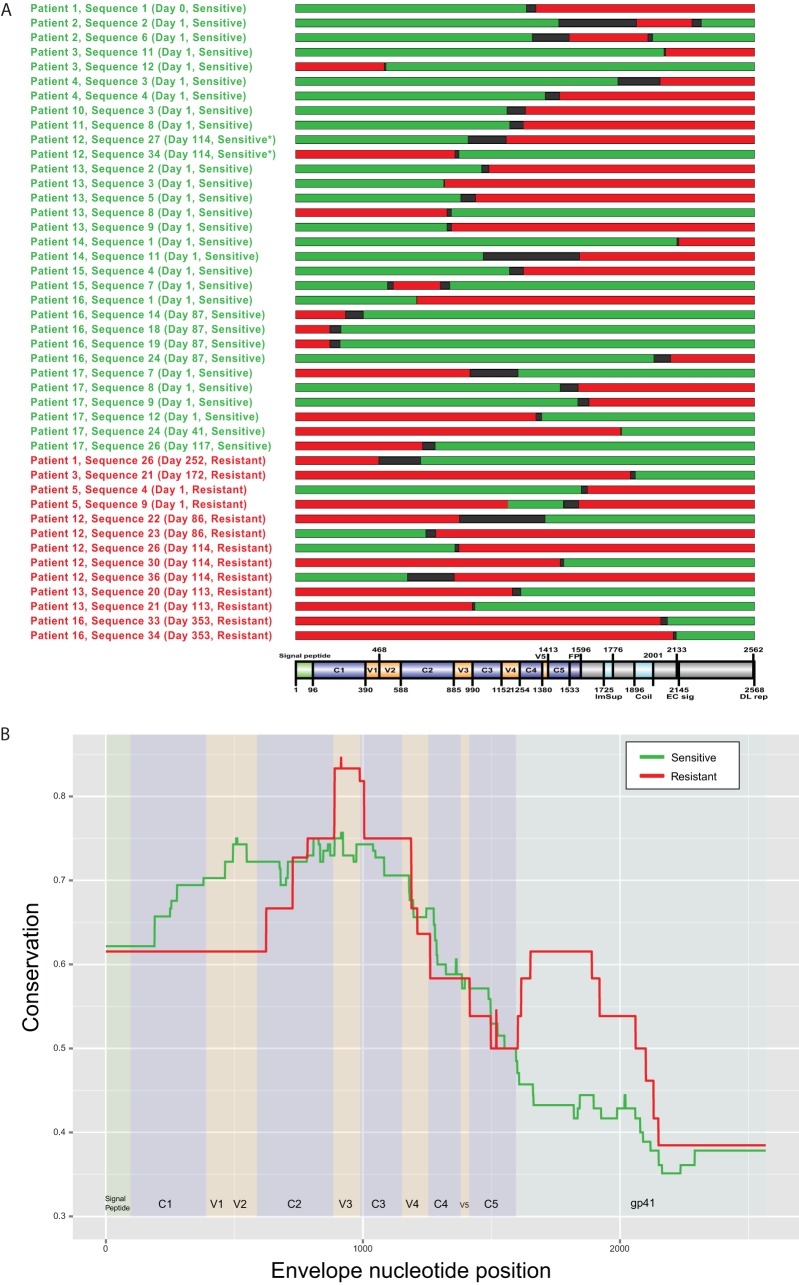
Mapping of recombination breakpoints for sensitive-resistant recombinants. (A) Intrapatient recombination between sensitive and resistant sequences was detected using Recco. The sequenced regions are mapped out relative to the HXB2 reference sequence, with a domain map showing the relative regions. For each sequence listed, the patient name and sequence number are indicated, as well as the day in therapy and R5 resistance, shown in parentheses. The colors indicate the parental sequence, with red indicating R5-resistant sequences and green indicating R5-sensitive sequences, separated by probable breakpoint regions, shown in black. *, sensitive according to unpooled MPI score. (B) Plot indicating the conservation of sensitive (green) and resistant (red) regions in recombinant sequences.

Recco identified 31 and 13 recombinant viruses that were associated with phenotypically sensitive or resistant viruses, respectively (Fig. 2A). Comparing these, we delimited the regions of the envelope that confer the respective phenotype by identifying the regions that showed the highest conservation in either the sensitive or resistant recombinants: V1 to C3 in sensitive and C2 to C3 in resistant sequences (Fig. 2B). This indicates that the Env C2 to C3 regions, including in particular the V3 loop region, have an influence on resistance, i.e., the relevant amino acids must be present in these regions of the recombinant to confer the respective phenotype. This recombination analysis result, coupled with the high number of mutations observed in V2 and V3 and in the C3 N terminus, indicates that structural shifts in these areas are most likely involved in HIV-1's use of drug-bound CCR5.

Mutations in both V2 and V3 have been previously implicated in coreceptor binding (40–45). As the C3 region is structurally proximal to the V3 loop, interactions between these domains may be important for coreceptor recognition or binding. However, unlike other cases of HIV-1 drug resistance, the sequence positions in Env associated with R5-tropic resistance differed considerably between patients. Indeed, no identifiable shared sites (or specific residue changes) were found to be consistently involved in resistance emergence across all patients. Though sites within V3 were found to be divergent based upon drug susceptibility in most patients, few of these sites overlapped across multiple patients. This suggests that the resistance mutations that enable the virus to use maraviroc-bound CCR5 are not independent and are constrained by the need to maintain the structure/function of the envelope protein.

### Coevolution networks within and between patients.

To investigate potential coevolutionary/compensatory changes in Env, we identified structurally and/or functionally important covarying sites in both gp120 and gp41, which form complicated covariation networks in both sensitive and resistant R5 viruses. In the coevolution analysis, we focused on covarying sites that could be associated with hydrophobicity and/or molecular weight covariation, as these two factors are among the most important for explaining amino acid contributions to protein structural stability (46).

We first analyzed data from individual patients and looked for common patterns that might be shared between patient data sets. To do this, we identified covarying sites within each of the patient data sets, combining sequences from the two time points: before treatment (sensitive) and after treatment (resistant). Eighteen patients had detectable covariation networks (see Fig. S3 in the supplemental material); the exceptions were patients 4 and 8. In the 18 covariation networks, there were covarying sites in both gp120 and gp41, except for three patients (5, 9, and 10). We observed seven patients (1, 2, 5, 9, 15, 16, and 17) with V3 loop involvement in their covariation networks (see Fig. S3 in the supplemental material). In one patient (patient 1), the signal peptide was involved in within-patient coevolution (see Fig. S3 in the supplemental material). Analysis of the sensitive and resistant viruses of individual patients indicated that there was no statistically significant difference between the sensitive and resistant viruses in terms of numbers of N-linked glycosylation sites (paired *t* test, df = 15, Pr(|*T*| > |*t*|) = 0.81). However, the location and number of glycosylation sites between sensitive and resistant viruses changed within some patients (patients 2, 5, 9, 11, 13, and 16). All the patients had their own unique covariation networks, indicating a patient-dependent coevolutionary profile of the viral population. In the placebo arm (patients 18, 19, and 20), there were no covarying sites involved in the V3 loop (see Fig. S3 in the supplemental material).

To further understand the evolution of maraviroc resistance in individual patients, we analyzed the sensitive and resistant sequences separately. Although we detected covarying sites in only two patients (patients 10 and 13), the results revealed two striking observations: the identified covarying sites were all in the vicinity of PNGMs, and the changes in hydrophobicity of these covarying pairs were negatively correlated in the sensitive viruses, while in the resistant viruses, they were positively correlated. Note that molecular weight changes were negatively correlated in both groups (see Table S7 in the supplemental material).

To identify between-patient patterns of coevolution, we next combined sensitive and resistant sequences from all 16 patients identified by their population dynamics (Fig. 2; see Fig. S2A and B in the supplemental material). The general pattern observed in individual patients was consistent with the global pattern seen between patients. Interestingly, considering covarying sites associated with hydrophobicity and molecular weight covariation, we found that both (sensitive and resistant) covariation networks were enriched with the same number of sites associated with potential PNGMs (15 sites in the sensitive network [χ^2^ = 51.80, *P* < 0.001; Fisher exact test, *P* < 0.001] and 15 sites in the resistant network [χ^2^ = 55.81, *P* < 0.001; Fisher exact test, *P* < 0.001]).

**FIG 3:**
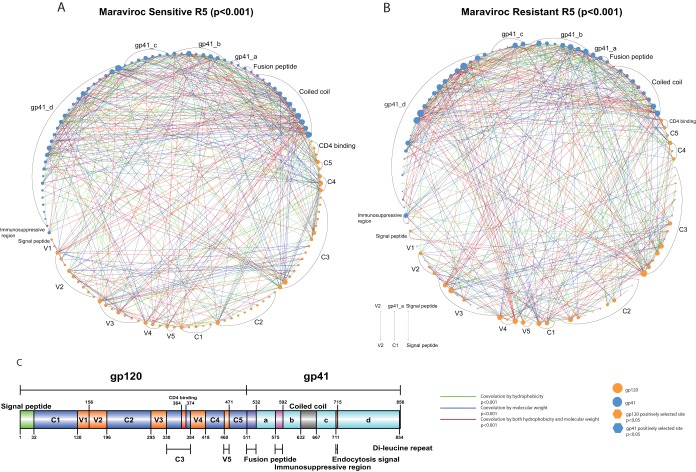
Covariation networks of gp120 and gp41 in sensitive and resistant R5 viruses. (A and B) Covariation networks of HIV-1 envelope protein associated with hydrophobicity and/or molecular weight covariation for maraviroc-sensitive (A) and -resistant (B) CCR5 viruses. (C) Covarying amino acid sites are organized and labeled by protein domains in gp120 (orange circles) and gp41 (blue circles). The circle sizes indicate the relative numbers of interactions in the covariation networks. Covarying sites are linked by colored lines, which indicate the physicochemical properties of covariation (see the key below and Table 1 for a summary). Sites under positive selection are shown as hexagons. Information regarding positive selection, protein domain, and glycosylation can be found in Tables S1 and S3 in the supplemental material for covarying sites in sensitive and resistant viruses, respectively.

In the sensitive R5 viruses, we identified a covariation network consisting of 130 amino acid sites forming 1,413 covarying pairs by BLOSUM score covariation (Table 1). This covariation network can be further refined by hydrophobicity and molecular weight covariation. One hundred three sites forming 295 covarying pairs (with a connection coefficient of 0.056) can be associated with hydrophobicity covariation, whereas 108 sites forming 319 covarying pairs (connection coefficient, 0.055) can be associated with molecular weight covariation (Table 1 and Fig. 3A). There were 81 sites forming 131 covarying pairs (connection coefficient, 0.040) that can be associated with both hydrophobicity and molecular weight covariation (Table 1 and Fig. 3A). Furthermore, there were 120 sites forming 483 covarying pairs (connection coefficient, 0.067) associated with hydrophobicity and/or molecular weight (Table 1 and Fig. 3A).

**TABLE 1:** Summary of the sensitive and resistant covariation networks^*a*^

Type	Covarying sites (nodes)		Covarying pairs (edges)	
	Sensitive	Resistant	Sensitive	Resistant
BLOSUM62	130	125	1,413	1,400
Hydrophobicity	103	99	295	305
Molecular wt	108	106	319	326
Hydrophobicity and molecular wt	81	77	131	147
Hydrophobicity/molecular wt/hydrophobicity and molecular wt	120	114	483	484

a The numbers of covarying sites and pairs for sensitive and resistant networks are summarized. Four types of networks were compared: covariation by BLOSUM62 score covariation, covariation by hydrophobicity and/or molecular weight covariation.

In the resistant R5 viruses, we identified a covariation network with 125 amino acid sites forming 1,400 covarying pairs in terms of BLOSUM score covariation (Table 1). After considering hydrophobicity and molecular weight, we found 99 sites forming 305 covarying pairs (connection coefficient, 0.063) with hydrophobicity covariation, and for molecular weight covariation, we found 106 sites forming 326 covarying pairs (connection coefficient, 0.058) (Table 1 and Fig. 3B). Combining both hydrophobicity and molecular weight, we identified 77 sites forming 147 covarying pairs (connection coefficient, 0.050) (Table 1 and Fig. 3B). There were 114 sites forming 484 covarying pairs associated with hydrophobicity and/or molecular weight covariation (connection coefficient, 0.075). Moreover, we found that there was positive correlation of hydrophobicity covariation (Spearman's ρ = 0.71, *P* < 0.0001) in the shared covariation network between sensitive and resistant sequences from all 20 patients (between-patient analysis). The molecular weight covariation was also positively correlated (Spearman's ρ = 0.86, *P* < 0.0001). Moreover, in the placebo arm (patients 18, 19, and 20), both hydrophobicity and molecular weight covariation were positively correlated between the two time points (Spearman's ρ = 0.82, *P* < 0.0001, and Spearman's ρ = 0.88, *P* < 0.0001, respectively).

This finding of similar numbers of sites covarying in the sensitive and resistant viral data sets indicates that compensatory, i.e., structurally linked, changes that maintain various functionalities are a normal part of envelope evolution. Envelope-mediated drug resistance is thus a specific evolutionary path among possible evolutionary trajectories. To confirm the importance of the identified covarying sites, we performed positive-selection analysis (after removing the detected recombinants mentioned above). In the sensitive R5 viruses, we identified 45 sites under positive selection, and 33 of these sites exhibited detectable coevolution (χ^2^ = 102.08, *P* < 0.00001), i.e., 17 sites in gp120 and 16 sites in gp41 (see Tables S1 and S2 in the supplemental material). Conversely, in the resistant R5 viruses, we identified 69 sites under positive selection, and 51 of these sites exhibited detectable coevolution (χ^2^ = 264.57, *P* < 0.00001), with 27 sites in gp120 and 24 sites in gp41 (see Tables S3 and S4 in the supplemental material). Interestingly, we observed that positively selected sites were enriched for PNGM motifs in both sensitive (6 sites; χ^2^ = 22.75, *P* < 0.001; Fisher exact test, *P* < 0.001) and resistant (8 sites; χ^2^ = 24.69, *P* < 0.001; Fisher exact test, *P* < 0.001) viruses.

To further validate our observations, we analyzed viruses from the three placebo-treated patients (18, 19, and 20) by combining their sequences from the first and second time points. We observed similar numbers of covarying sites before and after treatment—45 and 52 sites, respectively—which formed 256 and 795 covarying pairs and had connection coefficients of 0.259 and 0.599, respectively. This increase of covarying pairs was in contrast to the group of patients receiving maraviroc because, interestingly, the trend of change for the number of covarying pairs was higher in the placebo arm (Table 1), indicating stronger structural and/or functional constraints acting on the maraviroc-resistant populations and consistent with a genetic bottleneck in the emergence of R5-tropic resistance. After considering hydrophobicity and/or molecular weight, in addition to covarying sites identified by BLOSUM62 score covariation, we still observed similar numbers of covarying sites (45 and 50 sites), which formed 238 (see Fig. S4 and Table S5 in the supplemental material) and 702 (see Fig. S5 and Table S6 in the supplemental material) covarying pairs having connection coefficients of 0.240 and 0.573, respectively. Intriguingly, we observed only a few PNGMs (three and six PNGMs before and after treatment [placebo arm], χ^2^ = 3.13, *P* > 0.05; Fisher exact test, *P* > 0.05 and χ^2^ = 16.99, *P* < 0.001; Fisher exact test, *P* = 0.002, respectively) involved in the two covariation networks (see Tables S5 and S6 in the supplemental material), which suggests a potential role of N-linked glycosylation in the evolution of R5 maraviroc resistance.

Next, to understand the mutational pathways that may lead to the evolution of maraviroc resistance, we focused on the key steps that HIV-1 requires to enter host cells. First, we compared three covariation networks between sensitive and resistant R5 viruses in the group of patients who received maraviroc, which centered on CD4 binding, CCR5 binding (V3 loop), and the secretory pathway (signal peptide dependent, which plays an important role in controlling the expression level of envelope protein on the virion). Second, we analyzed the results from the placebo arm to further validate our results.

### Coevolution between CD4 binding sites and other regions of gp120 and gp41 reveals novel mutational pathways leading to maraviroc resistance.

**FIG 4:**
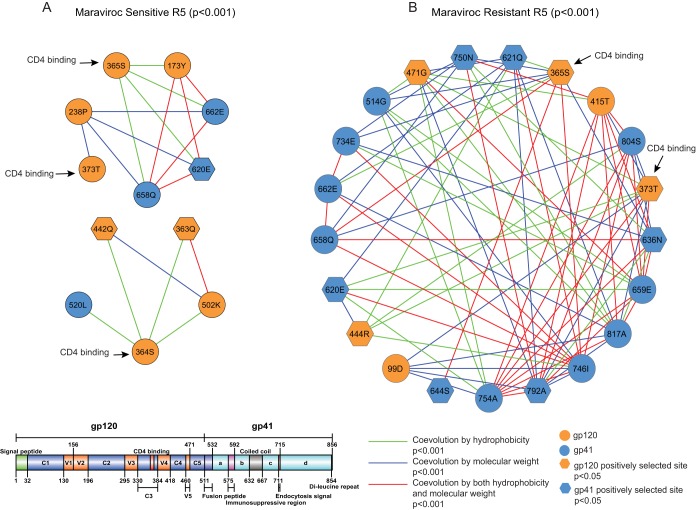
Covariation networks of CD4 binding sites. (A and B) Shown are covariation networks of the CD4 binding sites associated with hydrophobicity and/or molecular weight for sensitive (A) and resistant (B) R5 viruses. Covarying sites are mapped to the gp120 protein crystal structure. Sites under positive selection are shown as hexagons. Covarying sites are linked by colored lines indicating the nature of their covariation. The sizes of the circles indicate the relative numbers of interactions in the covariation networks (Fig. 3). Information regarding positive selection, protein domain, and glycosylation can be found in Tables 2 and 3 for covarying sites in sensitive and resistant viruses, respectively.

CD4 binding allows HIV-1 gp120 to bind to CCR5/CXCR4 and enter the host cell, which is a critical step in initiating the whole HIV infectious life cycle. In the sensitive R5 viruses, we found that 3 sites (364S, 365S, and 373T) in the CD4 binding region coevolved with 9 (5 in gp120 and 4 in gp41) sites forming a network with a connection coefficient of 0.288, while in the resistant viruses, we found that 2 sites (365S and 373T, with both under positive selection) from the CD4 binding region coevolved with 19 (4 in gp120 and 15 in gp41) sites forming a network with a connection coefficient of 0.409 (Fig. 4). In the sensitive R5 viruses, the five sites from gp120 were located in C2 (238P), C3 (363Q, under positive selection), C4 (442Q, under positive selection), C5 (502K), and V2 (173Y) (Fig. 4A). The other four sites from gp41 were located in the coiled-coil region (662E and 658Q), the fusion peptide (520L), and the gp41_b region (620E, under positive selection) (Fig. 4A). In the resistant R5 viruses, the four sites from gp120 were located in C1 (99D), C4 (444R, under positive selection), V4 (415T), and V5 (471G) (Fig. 4B). The other 15 sites from gp41 were located in the coiled-coil region (636N; 644S, under positive selection; 658Q; 662E; and 659E), the fusion peptide (514G), gp41_b (620E, under positive selection, and 621Q, under positive selection), and gp41_d (734E; 746I; 750N; 754A; 792A, under positive selection; 804S, under positive selection; and 817A) (Fig. 4). Interestingly, we found that in the sensitive R5 viruses, two (364S and 365S) of the three CD4 binding sites and one site (363Q) from C3 were located in the same potential N-linked glycosylation motif (N-X-S/T-X) (Table 2) (χ^2^ = 2.51, *P* > 0.05; Fisher exact test, *P* > 0.05). Moreover, there were two sites (365S in the CD4 binding region and 817A in the gp41_d region) from the resistant R5 viruses located in different PNGMs (Table 3) (χ^2^ = 1.79, *P* > 0.05; Fisher exact test, *P* > 0.05). Only the two CD4 binding sites (365S and 373T) from the resistant viruses were under positive selection, and the covariation network from the resistant viruses was more highly connected than the network from the sensitive viruses (Fig. 4A), which indicates an important role of CD4 binding in the evolution of maraviroc resistance. In summary, we have identified three (χ^2^ = 8, *P* = 0.00468) (Table 2) and 10 (χ^2^ = 37.06, *P* < 0.00001) (Table 3) sites under positive selection in sensitive and resistant covariation networks, respectively.

**TABLE 2:** Covarying sites in the CD4 binding network for sensitive R5 viruses

HXB2^*a*^	Degree^*b*^	Selection^*c*^	Site^*d*^	Domain	Protein	N-Gly motif^*e*^
173Y	13	Null	151	V2	gp120	
238P	15	Null	211	C2	gp120	−3
363Q	17	0.998267	331	C3	gp120	N-**X**-S/T-X^*f*^
364S	4	Null	332	CD4 binding	gp120	N-X-**S/T**-X^*f*^
365S	4	Null	333	CD4 binding	gp120	N-X-S/T-**X**^*f*^
373T	1	Null	341	CD4 binding	gp120	
442Q	21	0.999411	392	C4	gp120	−2
502K	7	Null	446	C5	gp120	
520L	4	Null	464	Fusion peptide	gp41	
620E	24	1	564	gp41_b	gp41	
658Q	5	Null	602	Coiled coil	gp41	
662E	22	Null	606	Coiled coil	gp41	

a HXB2 numbering is used as a reference.

b Degree, number of covarying pairs with the site in the BLOSUM62 covariation network.

c Selection indicates the results of positive-selection analysis (at 95% posterior probability); null indicates no positive selection.

d Site indicates the position of the covarying site in the alignment.

e N-Gly motif, the position of the covarying site in the N-linked glycosylation motif N-X-S/T-X. The position is highlighted in boldface and underlined, and positive/negative numbers indicate the position of the covarying site relative to its closest N-X-S/T-X motif in the sequence. Negative (−) means the covarying site is located on the left side of the motif, while positive (+) means the site is located on the right side of the motif.

f The sites are in the same N-X-S/T-X motif.

**TABLE 3:** Covarying sites in the CD4 binding network for resistant R5 viruses

HXB2^*a*^	Degree^*b*^	Selection^*c*^	Site^*d*^	Domain	Protein	N-Gly motif^*e*^
99D	16	Null	98	C1	gp120	
365S	9	0.968434	333	CD4 binding	gp120	N-X-S/T-**X**
373T	10	0.986405	341	CD4 binding	gp120	
415T	17	Null	365	V4	gp120	
444R	15	0.999052	394	C4	gp120	
471G	14	0.972233	415	V5	gp120	
514G	13	Null	458	Fusion peptide	gp41	
620E	16	0.999999	564	gp41_b	gp41	+1
621Q	23	0.999999	565	gp41_b	gp41	+2
636N	22	0.971159	580	Coiled coil	gp41	−1
644S	10	0.999716	588	Coiled coil	gp41	+4
658Q	10	Null	602	Coiled coil	gp41	
659E	19	Null	603	Coiled coil	gp41	
662E	12	Null	606	Coiled coil	gp41	
734E	26	Null	678	gp41_d	gp41	
746I	24	Null	690	gp41_d	gp41	
750N	18	0.978824	694	gp41_d	gp41	
754A	27	Null	698	gp41_d	gp41	
792A	18	0.999976	736	gp41_d	gp41	
804S	17	Null	748	gp41_d	gp41	
817A	20	Null	761	gp41_d	gp41	N-**X**-S/T-X

a HXB2 numbering is used as a reference.

b Degree, number of covarying pairs with the site in the BLOSUM62 covariation network.

c Selection indicates the results of positive-selection analysis (at 95% posterior probability); null indicates no positive selection.

d Site indicates the position of the covarying site in the alignment.

e N-Gly motif, the position of the covarying site in the N-linked glycosylation motif N-X-S/T-X. The position is highlighted in boldface and underlined, and positive/negative numbers indicate the position of the covarying site relative to its closest N-X-S/T-X motif in the sequence. Negative (−) means the covarying site is located on the left side of the motif, while positive (+) means the site is located on the right side of the motif.

### Coevolution between the V3 loop and other regions of gp120 and gp41 is enriched with sites in the N-linked glycosylation motifs.

**FIG 5:**
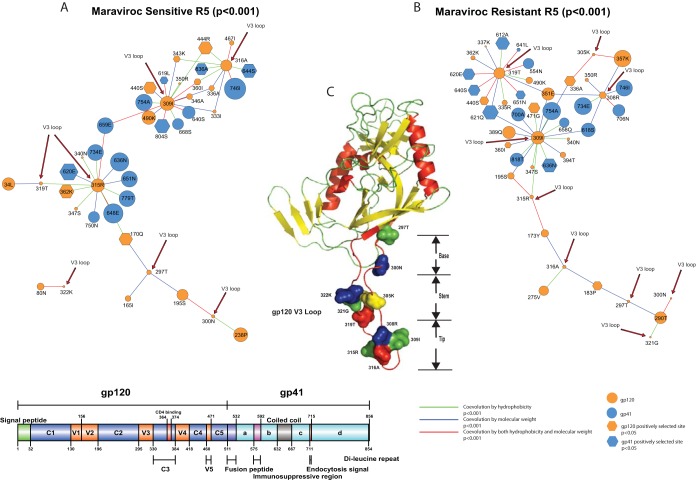
Covariation networks of the V3 loop. (A and B) Covariation networks of the HIV-1 envelope protein V3 loop associated with hydrophobicity and/or molecular weight for sensitive (A) and resistant (B) R5 viruses. (C) Covarying sites in the V3 loop mapped to the gp120 protein crystal structure. Sites under positive selection are shown as hexagons. Covarying sites are linked by colored lines indicating the nature of their covariation. The sizes of the circles indicate the relative numbers of interactions in the covariation networks (Fig. 3). Information regarding positive selection, protein domain, and glycosylation can be found in Tables 2 and 3 for covarying sites in sensitive and resistant viruses, respectively.

Among the sensitive R5 viruses, we identified 7 covarying sites in the V3 loop that coevolved with 19 sites in gp120 and 16 sites in gp41; this network had a connection coefficient of 0.051 (Fig. 5A and Table 4). Among the 19 covarying sites in gp120, sites were identified in C1 to C5 and V2, with sites in C3, C4, and V2 detected to be under positive selection (*P* < 0.05) (Fig. 5A and Table 4). Among the 16 covarying sites in gp41, there were 6 sites from the coiled-coil region (1 positively selected site) and 10 sites from the rest of gp41, with 3 positively selected sites identified (Fig. 5A and Table 4).

**TABLE 4:** Covarying sites in the V3 loop network for sensitive R5 viruses

HXB2 site^*a*^	Degree^*b*^	Selection^*c*^	Domain^*d*^	Protein	Site	N-Gly motif^*e*^
34L	12	Null	C1	gp120	33	
80N	5	Null	C1	gp120	79	
238P	15	Null	C2	gp120	211	−3
333I	2	Null	C3	gp120	305	N-**X**-S/T-X
336A	4	0.999804	C3	gp120	308	+1
340N	1	0.988433	C3	gp120	312	N-**X**-S/T-X
343K	5	0.981324	C3	gp120	315	+1
346A	4	Null	C3	gp120	318	+4
347S	2	1	C3	gp120	319	+5
350R	1	Null	C3	gp120	322	−2
360I	5	0.999305	C3	gp120	328	+1
362K	17	0.997297	C3	gp120	330	**N**-X-S/T-X
440S	13	0.997212	C4	gp120	390	
444R	15	0.999696	C4	gp120	394	−4
490K	14	Null	C5	gp120	434	
165I	4	Null	V2	gp120	143	+2
170Q	14	0.962951	V2	gp120	148	
195S	9	Null	V2	gp120	168	−2
297T	3	Null	V3	gp120	270	N-X-**S/T**-X
300N	2	Null	V3	gp120	273	+2
309I	15	Null	V3	gp120	282	+4
315R	13	Null	V3	gp120	286	
316A	9	Null	V3	gp120	287	
319T	2	Null	V3	gp120	290	
322K	1	Null	V3	gp120	293	
467I	3	Null	V5	gp120	411	
636N	24	Null	Coiled coil	gp41	580	−1
640S	12	Null	Coiled coil	gp41	584	N-X-S/T-**X**
644S	18	0.997303	Coiled coil	gp41	588	+4
648E	26	Null	Coiled coil	gp41	592	
651N	19	Null	Coiled coil	gp41	595	
659E	19	Null	Coiled coil	gp41	603	
619L	6	0.999707	gp41_b	gp41	563	N-X-S/T-**X**
620E	24	1	gp41_b	gp41	564	+1
668S	9	Null	gp41_c	gp41	612	
734E	19	Null	gp41_d	gp41	678	
746I	30	Null	gp41_d	gp41	690	
750N	9	Null	gp41_d	gp41	694	
754A	20	Null	gp41_d	gp41	698	
779T	19	Null	gp41_d	gp41	723	
804S	18	0.999353	gp41_d	gp41	748	
836A	13	1	gp41_d	gp41	780	

a HXB2 numbering is used as a reference.

b Degree, number of covarying pairs with the site in the BLOSUM62 covariation network.

c Selection indicates the results of positive-selection analysis (at 95% posterior probability); null indicates no positive selection.

d Site indicates the position of the covarying site in the alignment.

e N-Gly motif, the position of the covarying site in the N-linked glycosylation motif N-X-S/T-X. The position is highlighted in boldface and underlined, and positive/negative numbers indicate the position of the covarying site relative to its closest N-X-S/T-X motif in the sequence. Negative (−) means the covarying site is located on the left side of the motif, while positive (+) means the site is located on the right side of the motif.

In the resistant R5 viruses, we observed 9 covarying sites from the V3 loop, which coevolved with 20 sites from gp120 and 16 sites from gp41; this network had a connection coefficient of 0.048 (Fig. 5B and Table 5). Among the 20 covarying sites from gp120, we identified 14 in the conserved regions of gp120 (specifically C2, C3, and C4) and sites in V2, V4, and V5. Among the 16 covarying sites in gp41, there were 5 sites in the coiled-coil region (Fig. 5B and Table 5). Of the 44 total covarying sites among the resistant R5 viruses, we identified 20 sites under positive selection (*P* < 0.05) (Fig. 5B and Table 5). Interestingly, there were two positively selected sites in the V3 loop, which were located on opposite sides in the tip of the V3 loop (Fig. 5C and Table 5).

**TABLE 5:** Covarying sites in the V3 loop network for resistant R5 viruses

HXB2 site^*a*^	Degree^*b*^	Selection^*c*^	Domain^*d*^	Protein	Site	N-Gly motif^*e*^
275V	10	Null	C2	gp120	248	−1
290T	15	Null	C2	gp120	263	
335R	5	0.98715	C3	gp120	307	N-X-S/T-**X**
336A	13	0.999229	C3	gp120	308	+1
337K	1	0.99455	C3	gp120	309	+2
340N	2	0.99799	C3	gp120	312	N-**X**-S/T-X
347S	3	1	C3	gp120	319	+5
350R	3	0.974134	C3	gp120	322	−2
351E	16	Null	C3	gp120	323	−1
357K	25	Null	C3	gp120	325	N-**X**-S/T-X
360I	6	0.999998	C3	gp120	328	+1
362K	4	0.988469	C3	gp120	330	**N**-X-S/T-X
440S	9	0.974215	C4	gp120	390	
490K	6	Null	C5	gp120	434	
173Y	7	Null	V2	gp120	151	
183P	10	0.963713	V2	gp120	161	
195S	6	Null	V2	gp120	168	
297T	2	Null	V3	gp120	270	N-X-**S/T**-X
300N	1	Null	V3	gp120	273	+2
305K	2	Null	V3	gp120	278	+1
308R	7	0.999995	V3	gp120	281	+4
309I	17	Null	V3	gp120	282	+5
315R	3	Null	V3	gp120	286	
316A	3	0.954869	V3	gp120	287	
319T	13	Null	V3	gp120	290	
321G	1	Null	V3	gp120	292	
389Q	16	Null	V4	gp120	357	N-X-S/T-**X**
394T	5	Null	V4	gp120	362	N-X-**S/T**-X
471G	14	0.972233	V5	gp120	415	
636N	22	0.971159	Coiled coil	gp41	580	−1
640S	15	0.999965	Coiled coil	gp41	584	N-X-S/T-**X**
641L	3	0.994541	Coiled coil	gp41	585	+1
651N	10	0.964727	Coiled coil	gp41	595	
658Q	10	Null	Coiled coil	gp41	602	
554N	12	Null	gp41_a	gp41	498	
612A	19	1	gp41_b	gp41	556	
618S	19	Null	gp41_b	gp41	562	N-X-**S/T**-X
620E	16	0.999999	gp41_b	gp41	564	+1
621Q	23	0.999999	gp41_b	gp41	565	+2
700A	18	Null	gp41_c	gp41	644	
706N	15	Null	gp41_c	gp41	650	
734E	26	Null	gp41_d	gp41	678	
746I	24	Null	gp41_d	gp41	690	
754A	27	Null	gp41_d	gp41	698	
818T	18	Null	gp41_d	gp41	762	N-X-**S/T**-X

a HXB2 numbering is used as a reference.

b Degree, number of covarying pairs with the site in the BLOSUM62 covariation network.

c Selection indicates the results of positive-selection analysis (at 95% posterior probability); null indicates no positive selection.

d Site indicates the position of the covarying site in the alignment.

e N-Gly motif, the position of the covarying site in the N-linked glycosylation motif N-X-S/T-X. The position is highlighted in boldface and underlined, and positive/negative numbers indicate the position of the covarying site relative to its closest N-X-S/T-X motif in the sequence. Negative (−) means the covarying site is located on the left side of the motif, while positive (+) means the site is located on the right side of the motif.

To further understand the evolution of drug resistance, we compared the numbers of covarying pairs established by the same sites in the V3 loop between sensitive and resistant viruses and found that two sites (315R and 319T) had dramatic changes in the number of linked changes between sensitive and resistant viruses. The interactions changed from 13 to 3 for 315R (Fig. 5A and Table 2) and 2 to 13 for 319T (Fig. 5B and Table 5) in sensitive and resistant networks, respectively. This suggests 319T may be important for R5 resistance. However, among the 13 interactions 319T established, 11 were under positive selection (Fig. 5B and Table 5), which indicates that linked/compensatory changes between the V3 loop and other protein regions are also important for developing maraviroc R5 drug resistance.

**FIG 6:**
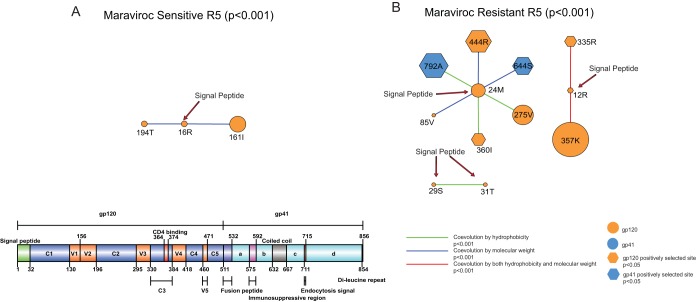
Covariation networks of the signal peptide. (A and B) Shown are covariation networks of the HIV-1 envelope protein signal peptide associated with hydrophobicity and/or molecular weight for sensitive (A) and resistant (B) R5 viruses. Sites under positive selection are shown as hexagons. Covarying sites are linked by colored lines indicating the nature of their covariation. The sizes of the circles indicate the relative numbers of interactions in the covariation networks (Fig. 3). Information regarding positive selection, protein domain, and glycosylation can be found in Tables 4 and 5 for covarying sites in sensitive and resistant viruses, respectively.

In the resistant R5 viruses, we observed that covarying sites located in the PNGM from the V3 loop covariation network were enriched (Table 5 and Fig. 5B) (10 sites; χ^2^ = 74.64, *P* < 0.001). However, the covarying sites from the sensitive virus were also enriched (Table 4 and Fig. 5A) (6 sites; χ^2^ = 25.09, *P* < 0.001; Fisher exact test, *P* < 0.001). Further inspection of these covarying sites in the resistant viruses located in the PNGM indicated that 7 of the 10 sites were located in gp120 (Table 5). Moreover, four sites were located in a conserved region of gp120 (C3), and three of these were detected to be under positive selection. There were three sites located in variable regions (1 in V3 and two in V4). Covarying sites located in the PNGM were also enriched in the conserved region (Table 5) (χ^2^ = 40.45, *P* < 0.001; Fisher exact test, *P* < 0.001). Besides the four sites located in the PNGM, we found there were seven covarying sites located in the vicinity of the PNGM (within five sites upstream or downstream of the N-X-S/T-X motif), with the exception of site 440S in C4 and 490K in C5 (Fig. 5B and Table 5). This indicates that all except two covarying sites in the conserved region of gp120 were associated with PNGMs (site 290T was predicted to be in a PNGM with a score, 0.49, below the threshold of 0.5) that coevolved with sites in the V3 loop. Similarly, in the V3 loop, we observed four sites (300N, 305K, 308R, and 309I) located in the vicinity of the PNGM. We found that covarying sites (357K and 335R) located in the PNGM also coevolved with site 12R in the signal peptide (Fig. 6B). In summary, we have identified 14 (χ^2^ = 57.41, *P* < 0.00001) (Table 4) and 20 (χ^2^ = 66.97, *P* < 0.00001) (Table 5) positively selected sites in sensitive and resistant covariation networks, respectively.

### Coevolution between the signal peptide and other regions of the envelope protein is enriched with sites in the N-linked glycosylation motifs in the resistant viruses.

Site 12R in the resistant viruses, but not in the sensitive viruses, was also under positive selection, suggesting the signal peptide plays a role in maraviroc resistance. Recently, mutational effects at position 12 in the signal peptide have been experimentally studied and shown to modulate the expression level of the envelope protein on the virion and viral infectivity (47). Some early studies also found that the signal peptide can control glycosylation and HIV-1's use of the secretory pathway (48, 49).

To understand the association between the signal peptide and maraviroc resistance, we analyzed the subnetwork formed between the signal peptide and other regions of the envelope protein. There were 3 and 12 covarying sites in the sensitive and resistant viruses, respectively; these networks had connection coefficients of 0.667 and 0.136 for sensitive and resistant networks, respectively (Fig. 6 and Tables 6 and 7). We found that the covariation network of the signal peptide in resistant viruses was enriched with sites in a PNGM (Table 7) (χ^2^ = 10.32, *P* < 0.01; Fisher exact test, *P* = 0.036). Interestingly, site 12R was involved only in the covariation network of resistant viruses (Tables 6 and 7), which was also positively selected (*P* < 0.01). Strikingly, as reported above, in the resistant viruses, 12R coevolved with sites 357K and 335R, which were located in the PNGM (Fig. 6B and Table 7). Moreover, all other sites that were not in the signal peptide also coevolved with sites from the V3 loop (Fig. 5B and 6B). Among these sites, 85V, 275V, 360I, and 444R in conserved regions of gp120 and site 644S from the coiled-coil region of gp41 were located in the vicinity of the PNGM (Table 7). We found that sites 85V, 335R, 360I, 444R, 644S, and 792A were also under positive selection (Table 7) (*P* < 0.05). These results from combining the resistance data indicate that the signal peptide indeed plays a role in HIV-1 N-linked glycosylation and the structure and/or function of the V3 loop, which can in turn have a role in the evolution of maraviroc resistance.

**TABLE 6:** Covarying sites in the signal peptide network for sensitive R5 viruses

HXB2 site^*a*^	Degree^*b*^	Selection^*c*^	Domain^*d*^	Protein	Site	N-Gly motif^*e*^
16R	2	Null	Signal peptide	gp120	15	
194T	7	Null	V2	gp120	167	−3
161I	2	Null	V2	gp120	139	

a HXB2 numbering is used as a reference.

b Degree, number of covarying pairs with the site in the BLOSUM62 covariation network.

c Selection indicates the results of positive-selection analysis (at 95% posterior probability); null indicates no positive selection.

d Site indicates the position of the covarying site in the alignment.

e N-Gly motif, the position of the covarying site in the N-linked glycosylation motif N-X-S/T-X. The negative number indicates the position of the covarying site relative to its closest N-X-S/T-X motif in the sequence. Negative (−) means the covarying site is located on the left side of the motif.

**TABLE 7:** Covarying sites in the signal peptide network for resistant R5 viruses

HXB2 site^*a*^	Degree^*b*^	Selection^*c*^	Domain^*d*^	Protein	Site	N-Gly motif^*e*^
85V	1	0.997736	C1	gp120	84	−3
275V	10	Null	C2	gp120	248	−1
335R	5	0.98715	C3	gp120	307	N-X-S/T-**X**
357K	25	Null	C3	gp120	325	N-**X**-S/T-X
360I	6	0.999998	C3	gp120	328	+1
444R	15	0.999052	C4	gp120	394	−4
12R	2	0.995959	Signal peptide	gp120	12	
24 M	6	Null	Signal peptide	gp120	23	
29S	1	Null	Signal peptide	gp120	28	
31T	1	Null	Signal peptide	gp120	30	
644S	10	0.999716	Coiled coil	gp41	588	+4
792A	18	0.999976	gp41_d	gp41	736	

a HXB2 numbering is used as a reference.

b Degree, number of covarying pairs with the site in the BLOSUM62 covariation network.

c Selection indicates the results of positive-selection analysis (at 95% posterior probability); null indicates no positive selection.

d Site indicates the position of the covarying site in the alignment.

e N-Gly motif, the position of the covarying site in the N-linked glycosylation motif N-X-S/T-X. The position is highlighted in boldface and underlined, and positive/negative numbers indicate the position of the covarying site relative to its closest N-X-S/T-X motif in the sequence. Negative (−) means the covarying site is located on the left side of the motif, while positive (+) means the site is located on the right side of the motif.

To validate the patterns observed from the V3 loop and the signal peptide covariation networks, we compared the results with those from the placebo arm. We found that these patterns were not observed in the placebo arm. Nevertheless, we observed two PNGMs in both the V3 loop (χ^2^ = 4.55, *P* < 0.05; Fisher exact test, *P* > 0.05) and the signal peptide (χ^2^ = 1.56, *P* > 0.05; Fisher exact test, *P* > 0.05) covariation networks before placebo treatment. After placebo treatment, we observed five and four PNGMs in the V3 loop (χ^2^ = 20.26, *P* < 0.001; Fisher exact test, *P* = 0.002) and the signal peptide (χ^2^ = 9.39, *P* < 0.01; Fisher exact test, *P* = 0.019) networks (see Tables S5 and S6 in the supplemental material). This evidence supports the view that N-linked glycosylation is important for V3 loop function (50) and that the signal peptide is crucial for both normal evolution of HIV-1 and entry inhibitor resistance (47, 51). In summary, we found no sites under positive selection in the sensitive covariation network (Table 6), whereas there were seven positively selected sites in the resistant covariation network (Table 7).

Finally, to understand how positively selected sites unique to maraviroc-resistant virus can contribute to resistance evolution, we constructed a covarying network of positively selected sites unique to resistant virus (see Fig. S6 and Table S8 in the supplemental material). Interestingly, key sites unique to resistant virus, as reported above (including CD4 binding, V3 loop, and signal peptide regions; N-linked glycans; the gp41 fusion peptide; and other gp41 regions) were found in this network (see Table S8 in the supplemental material). To understand the functional implications of these unique sites, we mapped them into a hypothetical structural complex, CD4-GP120-CCR5, with two PNGMs (279D and 365S, also positively selected covarying sites) visualized in Fig. S7 in the supplemental material. Intriguingly, when we mapped the sites to the complex, all the sites (excluding one, 92N), contributed to functionality: CD4 binding (350R, 279N, 467I, 471G, 365S, and 373T), N-linked glycosylation (279N and 365S), and CCR5 binding (316A) (see Fig. S7 in the supplemental material).

## DISCUSSION

In this study, we detected significant patterns of genetic change in the HIV-1 envelope that confer R5-tropic maraviroc resistance. We found that resistant sequences formed a new and distinct cluster, which was associated with a bottleneck occurring after therapy started, consistent with the resistant population most likely emerging *de novo*, i.e., as a result of the selective pressure provided by the drug, which leads to significantly more amino acid sites under positive selection and higher connection coefficients of the resistant virus covariation networks. Note that while we cannot discount a preexisting variant being resistant by chance and emerging under drug pressure, as reported in another CCR5 antagonist, aplaviroc (APL) (52), our results support the absence of any preexisting virus population of any significance.

We found that gp41 was involved in all the covariation networks and in individual patients, in particular, the covariation networks of CD4 binding, the V3 loop, and the signal peptide (not observed in the sensitive viruses), indicating its importance in R5 resistance evolution (53). Interestingly, we identified three sites (514, 515, and 520) located in the gp41 fusion peptide that may contribute to resistance, which are very close to three sites (516, 518, and 519) identified previously in another CCR5 antagonist, vicriviroc (54). Intriguingly, site 514 covaries with sites in the CD4 binding region in the resistant virus, but none of the three sites we identified covaries with any site in the V3 loop and signal peptide. This suggests the fusion peptide can contribute to developing CCR5 antagonist resistance in general, which is V3 independent. Moreover, there was a unique CD4 binding covariation network independent of the V3 loop but dependent on the gp41 fusion peptide contributing to the evolution of R5 maraviroc resistance, which is consistent with a previous report that there might be unique CD4 binding-dependent mutational pathways leading to the emergence of maraviroc resistance without the requirement for V3 loop mutations (12), possibly by binding more strongly to the CD4 coreceptor through coevolutionary changes in non-V3 regions and the fusion peptide. This suggests a potential treatment strategy that uses both entry and fusion inhibitors (such as enfuvirtide [55]) to minimize the chance for emergence of V3-independent CCR5 antagonist resistance.

N-linked glycosylation and signal peptide involvement further constrained the emergence of envelope-associated drug resistance. Collectively, these results demonstrate that while envelope sequence mutations do confer R5-tropic maraviroc resistance, the specific changes involved are largely context dependent, i.e., they are dependent on the genetic variation present in the patient's individual infection and thus are difficult to predict. This is a consequence of the malleable nature of HIV-1's envelope, which results in distinct constraints, so that different mutations are required to confer resistance in different infections. Therefore, appropriate coevolutionary/compensatory changes of important amino acid residues are vital for the maintenance of functional viral proteins. HIV-1's envelope, as a consequence, presents itself as a good drug target (in terms of a low propensity to evolve resistance) accounting for the uncommon observation of maraviroc resistance in R5-tropic virus on failure in clinical trials.

What is crucial to appreciate is that HIV-1 envelope glycosylation and protein folding are not independent, and this subjects Env to additional structural constraints that impact potential drug resistance pathways. The heavy glycosylation of gp120 (about 55% of its molecular mass is contributed by N-linked glycans [56]) facilitates folding of the Env polypeptide chain into its correct three-dimensional conformation, which stabilizes the protein and is vital for cell entry by the virus (17, 50, 57–60). It also acts as a “glycan shield” to protect the virion from neutralizing antibodies (56, 61). Moreover, our novel results suggest that coevolutionary changes between the signal peptide, N-linked glycosylation, and other functional domains of gp120/gp41 (especially the V3 loop) modulate envelope protein expression efficiency and glycosylation patterns. Note that expression differences associated with variants can also change without signal peptide involvement. These changes help the virus adapt to the host environment under “normal” immune pressure and during the emergence of drug resistance, such as we have investigated here. Although it is not clear how the signal peptide affects N-linked glycosylation, previous research has shown that oligosaccharides (glycans) are added to the translocating peptide cotranslationally, suggesting the signal peptide may control the glycosylation process by interacting with amino acids flanking the N-linked glycosylation sites/motifs in the envelope protein (57, 62).

The signal peptide of the HIV-1 envelope protein has been shown to determine the expression level of the glycosylated Env on the virion (47) and potentially determines vaccine efficacy (63). As the expression levels of the host coreceptors CCR5 and/or CXCR4 also fluctuate between cell lines (64), dynamic Env expression on the virion surface regulated by the signal peptide may confer great selective advantage on HIV populations (53). This can also be true for using the maraviroc-bound CCR5 for cell entry of R5-tropic viruses (65). The signal peptide plays a vital role in protein subcellular localization and function. The mutational effects of key amino acid substitutions in both the general secretion (Sec) pathway and the signal peptide have been extensively studied in bacteria. These studies found that mutations in both the secretion system and the signal peptide can affect protein secretion (62). More specifically, changes of charged residues in the signal peptide of the N-terminal end can increase or decrease the secretion rates of the underlying proteins (49). HIV envelope protein is secreted through the host Sec pathway and N-glycosylated cotranslationally (57). Moreover, the viral envelope signal peptide has to satisfy several constraints of the Sec pathway and the viral life cycle, which is only possible through extensive coevolutionary changes or compensatory mutations. First, in order to be secreted through the host Sec pathway, the viral signal peptide has to present a configuration similar to that of a typical host secreted protein (66). Therefore, the viral signal peptide does not deviate from the basic configuration of a eukaryotic signal peptide (67). What is unique to HIV-1 is a higher negative charge in the N-terminal region of the signal peptide, which is suggested to play a role in gp120 secretion efficiency (48). Second, virus-specific changes in the signal peptide are required throughout its life cycle (47). Third, the HIV signal peptide also determines gp120 glycosylation, which is critical for envelope structure and/or function.

In conclusion, R5 maraviroc resistance, mediated via CD4 binding/V3 loop mutations in the context of complex conformational changes, requires additional linked changes in gp120 and gp41 in order to maintain essential functionalities, while changes in glycosylation patterns are tied to conformational changes and are intrinsically linked to protein stability. In addition, maintenance of optimal gp120 expression and efficiency is important, as exemplified here by the finding of signal peptide involvement in the facilitation of maraviroc resistance. HIV-1's envelope protein thus presents itself as a drug target with an inherent impediment to resistance evolution accounting for the low rate of occurrence of R5-tropic maraviroc-resistant virus observed.

## Supplementary Material

Supplemental material
