# Supplementary material for: Characterizing the Diverse Mutational Pathways Associated with R5-Tropic Maraviroc Resistance: HIV-1 That Uses the Drug-Bound CCR5 Coreceptor
Source: J Virol. 2015 Sep 2;89(22):11457–72. doi: 10.1128/JVI.01384-15 (PMC4645647; doi:10.1128/JVI.01384-15)
Supplement: Supplemental material [file supp_89_22_11457__index.html]

Supplemental material 

# Characterising the diverse mutational pathways associated with R5-tropic maraviroc resistance: HIV-1 that uses the drug-bound CCR5 coreceptor

## Supplemental material

- Supplemental file 1 -

  Fig. S1 (Plot of luciferase-generated relative light units for CCR5 entry efficiency.)

  Fig. S2 (Phylogenetic trees for maraviroc patients 1 to 8 and 9 to16 and placebo-arm patients 18 to 20.)

  Fig. S3 (Covariation networks of virus from 18 patients.)

  Fig. S4 (Covariation network from viruses when placebo-arm treatment started.)

  Fig. S5 (Covariation network from viruses after placebo-arm treatment.)

  Fig. S6 (A network of covarying sites under positive selection unique to maraviroc-resistant virus.)

  Fig. S7 (Coevolving sites under positive selection unique to resistant virus mapped to a hypothetical CD4-GP120-CCR5 structural complex.)

  PDF, 2.2M
- Supplemental file 2 -

  Table S1 (Coevolving sites from sensitive viruses (patients 1 to 16).)

  Table S2 (Positively selected sites from all sensitive viruses (patients 1 to 20).)

  Table S3 (Coevolving sites from resistant viruses (patients 1 to 16).)

  Table S4 (Positively selected sites from all resistant viruses.)

  Table S5 (Coevolving sites from viruses when placebo-arm treatment started.)

  Table S6 (Coevolving sites from viruses after placebo-arm treatment. )

  Table S7 (Coevolving sites for patients 10 and 13.)

  Table S8 (Positively selected coevolving sites unique to resistant viruses (patients 1 to 16).)

  XLS, 115K
