# Supplementary material for: Characterizing the Diverse Mutational Pathways Associated with R5-Tropic Maraviroc Resistance: HIV-1 That Uses the Drug-Bound CCR5 Coreceptor
Source: J Virol. 2015 Sep 2;89(22):11457–72. doi: 10.1128/JVI.01384-15 (PMC4645647; doi:10.1128/JVI.01384-15)
Supplement: Supplemental material [file JVI.01384-15_zjv999090957so1.pdf]

Supplementary Figures:

Figure S1. Plot of luciferase-generated relative light units (RLU) for CCR5 entry efficiency. Clone MPI values are plotted against CCR5 (R5) and CXCR4 (X4) RLU. Blue dots represent CCR5 using clones, while red dots represent CXCR4 using clones. Plots are fitted with linear regression for R5 RLU and X4 RLU, respectively. The results indicate that regardless of resistance state all these clones are R5-tropic viruses.

Figure S1

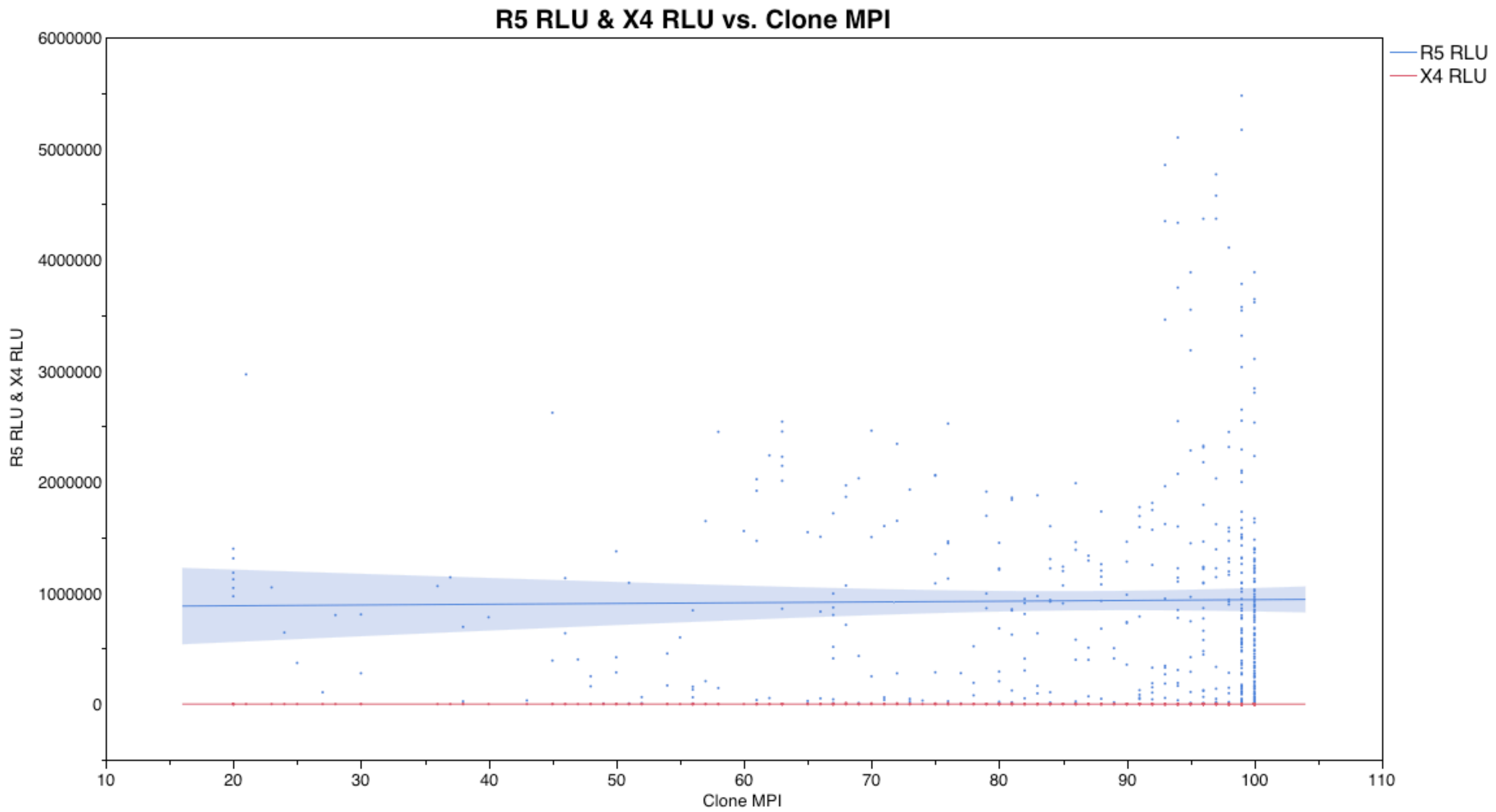
R5 RLU & X4 RLU vs. Clone MPI
R5 RLU & X4 RLU
Clone MPI
R5 RLU
X4 RLU
6000000
5000000
4000000
3000000
2000000
1000000
0
10
20
30
40
50
60
70
80
90
100
110

Figure S2. Phylogenetic trees for maraviroc patients (A) 1-8, (B) 9- 16, and placebo-arm patients (C) 18-20. Patient 17 was omitted from phylogenetic analysis due to a lack of scores of pooled resistant MPI. Phylogenetic trees were created using BEAST v1.8.1, with tip nodes calibrated by days since therapy began. Tip nodes show patient number and sequence number, along with sampling day, pooled MPI, and sequence MPI shown in brackets. Node labels are colored by pooled MPI, with red indicating resistant strains (<95% MPI) and green indicating sensitive strains. To the right of every tree, Bayesian skyline plots (top right) and viral load plots (bottom right) are shown. Bayesian skyline plots (black lines) indicate inferred genetic diversity over time (in days) since therapy commencement, which is indicated by a grey vertical line; the blue lines represent the 95% highest posterior density confidence intervals. Viral load plots show the amount of HIV-1 RNA per ml of plasma, with samples taken at multiple time-points, before therapy commencement, during therapy and after therapy termination.

Figure S2 A

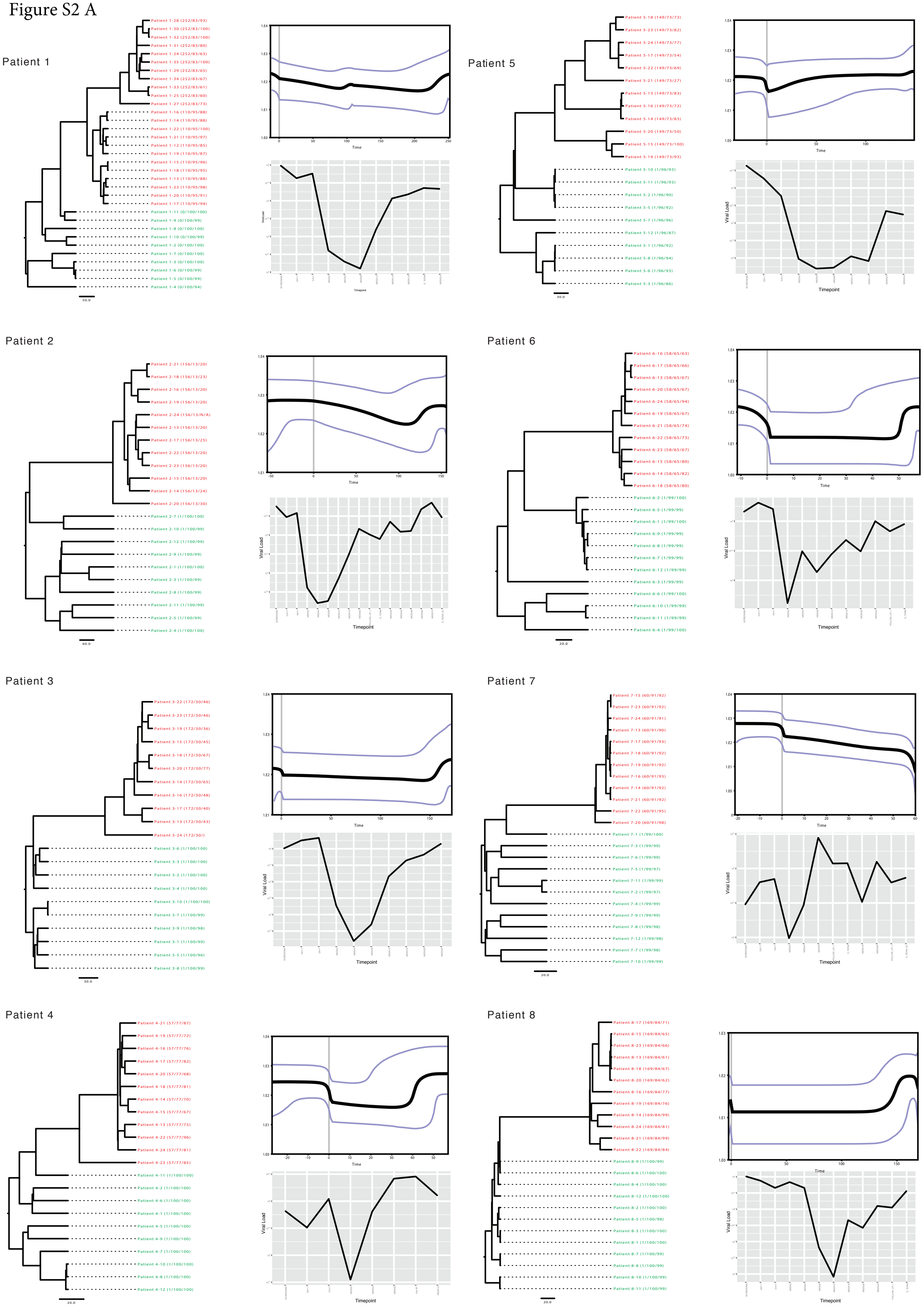

Patient 1
Patient 1-28 (252/83/93)
Patient 1-30 (252/83/100)
Patient 1-32 (252/83/100)
Patient 1-31 (252/83/80)
Patient 1-24 (252/83/63)
Patient 1-35 (252/83/100)
Patient 1-29 (252/83/65)
Patient 1-34 (252/83/67)
Patient 1-33 (252/83/61)
Patient 1-25 (252/83/60)
Patient 1-27 (252/83/73)
Patient 1-16 (110/95/88)
Patient 1-14 (110/95/88)
Patient 1-22 (110/95/100)
Patient 1-21 (110/95/97)
Patient 1-12 (110/95/85)
Patient 1-19 (110/95/87)
Patient 1-15 (110/95/96)
Patient 1-18 (110/95/95)
Patient 1-13 (110/95/88)
Patient 1-23 (110/95/98)
Patient 1-20 (110/95/91)
Patient 1-17 (110/95/94)
Patient 1-11 (0/100/100)
Patient 1-9 (0/100/99)
Patient 1-8 (0/100/100)
Patient 1-10 (0/100/99)
Patient 1-2 (0/100/100)
Patient 1-7 (0/100/100)
Patient 1-3 (0/100/100)
Patient 1-6 (0/100/99)
Patient 1-5 (0/100/99)
Patient 1-4 (0/100/94)
50.0
Time
Timepoint
Viral Load
Patient 5
Patient 5-18 (149/73/73)
Patient 5-23 (149/73/82)
Patient 5-24 (149/73/77)
Patient 5-17 (149/73/54)
Patient 5-22 (149/73/69)
Patient 5-21 (149/73/27)
Patient 5-13 (149/73/83)
Patient 5-16 (149/73/72)
Patient 5-14 (149/73/83)
Patient 5-20 (149/73/50)
Patient 5-15 (149/73/100)
Patient 5-19 (149/73/93)
Patient 5-10 (1/96/93)
Patient 5-11 (1/96/93)
Patient 5-2 (1/96/90)
Patient 5-5 (1/96/92)
Patient 5-7 (1/96/96)
Patient 5-12 (1/96/87)
Patient 5-1 (1/96/92)
Patient 5-8 (1/96/94)
Patient 5-6 (1/96/93)
Patient 5-3 (1/96/86)
30.0
Time
Timepoint
Viral Load
Patient 2
Patient 2-21 (156/13/20)
Patient 2-18 (156/13/23)
Patient 2-16 (156/13/20)
Patient 2-19 (156/13/20)
Patient 2-24 (156/13/N/A)
Patient 2-13 (156/13/20)
Patient 2-17 (156/13/25)
Patient 2-22 (156/13/20)
Patient 2-23 (156/13/20)
Patient 2-15 (156/13/20)
Patient 2-14 (156/13/24)
Patient 2-20 (156/13/30)
Patient 2-7 (1/100/100)
Patient 2-10 (1/100/99)
Patient 2-12 (1/100/99)
Patient 2-9 (1/100/99)
Patient 2-1 (1/100/100)
Patient 2-3 (1/100/99)
Patient 2-8 (1/100/99)
Patient 2-11 (1/100/99)
Patient 2-5 (1/100/99)
Patient 2-4 (1/100/100)
60.0
Time
Timepoint
Viral Load
Patient 6
Patient 6-16 (58/65/63)
Patient 6-17 (58/65/66)
Patient 6-13 (58/65/67)
Patient 6-20 (58/65/67)
Patient 6-24 (58/65/94)
Patient 6-19 (58/65/67)
Patient 6-21 (58/65/74)
Patient 6-22 (58/65/73)
Patient 6-23 (58/65/67)
Patient 6-15 (58/65/80)
Patient 6-14 (58/65/82)
Patient 6-18 (58/65/80)
Patient 6-2 (1/99/100)
Patient 6-5 (1/99/99)
Patient 6-1 (1/99/100)
Patient 6-9 (1/99/99)
Patient 6-8 (1/99/99)
Patient 6-7 (1/99/99)
Patient 6-12 (1/99/99)
Patient 6-3 (1/99/99)
Patient 6-6 (1/99/100)
Patient 6-10 (1/99/99)
Patient 6-11 (1/99/99)
Patient 6-4 (1/99/100)
20.0
Time
Timepoint
Viral Load
Patient 3
Patient 3-22 (172/30/46)
Patient 3-23 (172/30/46)
Patient 3-19 (172/30/36)
Patient 3-15 (172/30/45)
Patient 3-18 (172/30/67)
Patient 3-20 (172/30/77)
Patient 3-14 (172/30/65)
Patient 3-16 (172/30/48)
Patient 3-17 (172/30/40)
Patient 3-13 (172/30/43)
Patient 3-24 (172/30/)
Patient 3-6 (1/100/100)
Patient 3-3 (1/100/100)
Patient 3-2 (1/100/100)
Patient 3-4 (1/100/100)
Patient 3-10 (1/100/100)
Patient 3-7 (1/100/99)
Patient 3-9 (1/100/98)
Patient 3-1 (1/100/99)
Patient 3-5 (1/100/96)
Patient 3-8 (1/100/99)
30.0
Time
Timepoint
Viral Load
Patient 7
Patient 7-15 (60/91/92)
Patient 7-23 (60/91/92)
Patient 7-24 (60/91/91)
Patient 7-13 (60/91/90)
Patient 7-17 (60/91/93)
Patient 7-18 (60/91/92)
Patient 7-19 (60/91/92)
Patient 7-16 (60/91/93)
Patient 7-14 (60/91/92)
Patient 7-21 (60/91/92)
Patient 7-22 (60/91/95)
Patient 7-20 (60/91/98)
Patient 7-1 (1/99/100)
Patient 7-3 (1/99/99)
Patient 7-6 (1/99/99)
Patient 7-5 (1/99/97)
Patient 7-11 (1/99/99)
Patient 7-2 (1/99/97)
Patient 7-4 (1/99/99)
Patient 7-9 (1/99/99)
Patient 7-8 (1/99/98)
Patient 7-12 (1/99/98)
Patient 7-7 (1/99/98)
Patient 7-10 (1/99/99)
20.0
Time
Timepoint
Viral Load
Patient 4
Patient 4-21 (57/77/87)
Patient 4-19 (57/77/72)
Patient 4-16 (57/77/76)
Patient 4-17 (57/77/82)
Patient 4-20 (57/77/68)
Patient 4-18 (57/77/81)
Patient 4-14 (57/77/70)
Patient 4-15 (57/77/67)
Patient 4-13 (57/77/75)
Patient 4-22 (57/77/96)
Patient 4-24 (57/77/81)
Patient 4-23 (57/77/85)
Patient 4-11 (1/100/100)
Patient 4-2 (1/100/100)
Patient 4-6 (1/100/100)
Patient 4-1 (1/100/100)
Patient 4-5 (1/100/100)
Patient 4-9 (1/100/100)
Patient 4-7 (1/100/100)
Patient 4-10 (1/100/100)
Patient 4-8 (1/100/100)
Patient 4-12 (1/100/100)
20.0
Time
Timepoint
Viral Load
Patient 8
Patient 8-17 (169/84/71)
Patient 8-15 (169/84/65)
Patient 8-23 (169/84/66)
Patient 8-13 (169/84/61)
Patient 8-18 (169/84/67)
Patient 8-20 (169/84/62)
Patient 8-16 (169/84/77)
Patient 8-19 (169/84/76)
Patient 8-14 (169/84/99)
Patient 8-24 (169/84/81)
Patient 8-21 (169/84/99)
Patient 8-22 (169/84/84)
Patient 8-9 (1/100/99)
Patient 8-6 (1/100/100)
Patient 8-4 (1/100/100)
Patient 8-12 (1/100/100)
Patient 8-2 (1/100/100)
Patient 8-5 (1/100/98)
Patient 8-3 (1/100/100)
Patient 8-1 (1/100/100)
Patient 8-7 (1/100/99)
Patient 8-8 (1/100/99)
Patient 8-10 (1/100/99)
Patient 8-11 (1/100/99)
20.0
Time
Timepoint
Viral Load

Figure S2 B

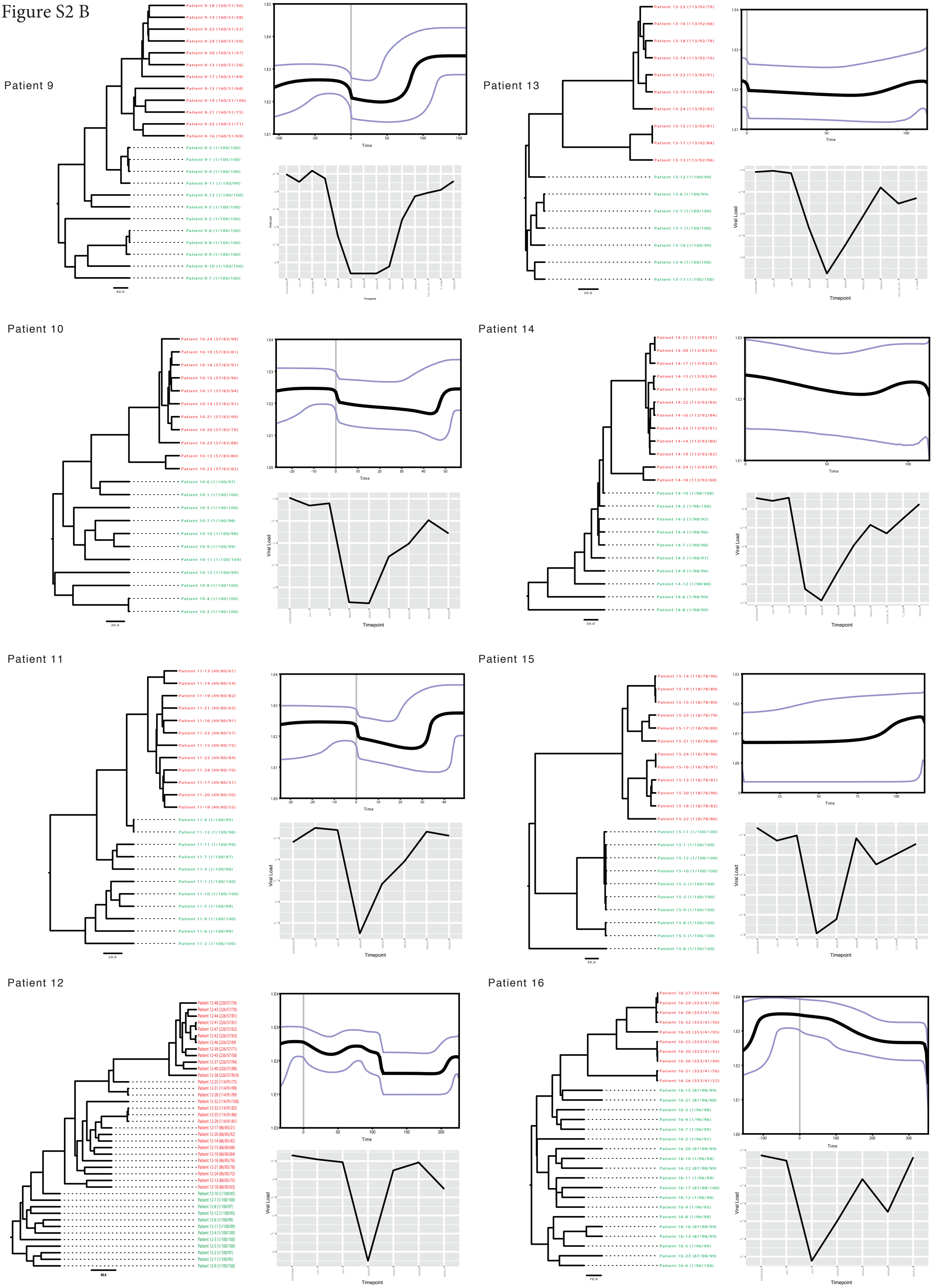

Patient 9
Patient 9-18 (160/51/30)
Patient 9-14 (160/51/38)
Patient 9-22 (160/51/52)
Patient 9-24 (160/51/50)
Patient 9-20 (160/51/47)
Patient 9-13 (160/51/28)
Patient 9-17 (160/51/49)
Patient 9-15 (160/51/68)
Patient 9-19 (160/51/100)
Patient 9-21 (160/51/75)
Patient 9-23 (160/51/71)
Patient 9-16 (160/51/69)
Patient 9-3 (1/100/100)
Patient 9-1 (1/100/100)
Patient 9-4 (1/100/100)
Patient 9-11 (1/100/99)
Patient 9-12 (1/100/100)
Patient 9-5 (1/100/100)
Patient 9-2 (1/100/100)
Patient 9-6 (1/100/100)
Patient 9-8 (1/100/100)
Patient 9-9 (1/100/100)
Patient 9-10 (1/100/100)
Patient 9-7 (1/100/100)
40.0
Time
Timepoint
Patient 13
Patient 13-23 (113/92/79)
Patient 13-16 (113/92/66)
Patient 13-18 (113/92/78)
Patient 13-14 (113/92/76)
Patient 13-22 (113/92/91)
Patient 13-19 (113/92/94)
Patient 13-24 (113/92/92)
Patient 13-15 (113/92/81)
Patient 13-17 (113/92/84)
Patient 13-13 (113/92/96)
Patient 13-12 (1/100/99)
Patient 13-6 (1/100/99)
Patient 13-7 (1/100/100)
Patient 13-1 (1/100/100)
Patient 13-10 (1/100/99)
Patient 13-4 (1/100/100)
Patient 13-11 (1/100/100)
20.0
Time
Viral Load
Timepoint
Patient 10
Patient 10-24 (57/83/99)
Patient 10-19 (57/83/81)
Patient 10-16 (57/83/91)
Patient 10-15 (57/83/96)
Patient 10-17 (57/83/94)
Patient 10-14 (57/83/91)
Patient 10-21 (57/83/99)
Patient 10-20 (57/83/79)
Patient 10-22 (57/83/88)
Patient 10-13 (57/83/80)
Patient 10-23 (57/83/82)
Patient 10-6 (1/100/97)
Patient 10-1 (1/100/100)
Patient 10-5 (1/100/100)
Patient 10-7 (1/100/98)
Patient 10-10 (1/100/98)
Patient 10-9 (1/100/99)
Patient 10-11 (1/100/100)
Patient 10-12 (1/100/99)
Patient 10-8 (1/100/100)
Patient 10-4 (1/100/100)
Patient 10-2 (1/100/100)
20.0
Time
Viral Load
Timepoint
Patient 14
Patient 14-21 (113/92/91)
Patient 14-20 (113/92/92)
Patient 14-17 (113/92/87)
Patient 14-13 (113/92/94)
Patient 14-15 (113/92/92)
Patient 14-22 (113/92/84)
Patient 14-16 (113/92/84)
Patient 14-23 (113/92/91)
Patient 14-14 (113/92/80)
Patient 14-19 (113/92/83)
Patient 14-24 (113/92/87)
Patient 14-18 (113/92/68)
Patient 14-10 (1/98/100)
Patient 14-2 (1/98/100)
Patient 14-3 (1/98/95)
Patient 14-4 (1/98/96)
Patient 14-7 (1/98/98)
Patient 14-5 (1/98/97)
Patient 14-9 (1/98/96)
Patient 14-12 (1/98/80)
Patient 14-6 (1/98/99)
Patient 14-8 (1/98/99)
30.0
Time
Viral Load
Timepoint
Patient 11
Patient 11-13 (49/80/61)
Patient 11-14 (49/80/54)
Patient 11-19 (49/80/82)
Patient 11-21 (49/80/63)
Patient 11-16 (49/80/91)
Patient 11-23 (49/80/57)
Patient 11-15 (49/80/75)
Patient 11-22 (49/80/84)
Patient 11-24 (49/80/70)
Patient 11-17 (49/80/51)
Patient 11-20 (49/80/50)
Patient 11-18 (49/80/55)
Patient 11-4 (1/100/95)
Patient 11-12 (1/100/96)
Patient 11-11 (1/100/99)
Patient 11-7 (1/100/97)
Patient 11-3 (1/100/96)
Patient 11-1 (1/100/100)
Patient 11-10 (1/100/100)
Patient 11-5 (1/100/99)
Patient 11-9 (1/100/100)
Patient 11-6 (1/100/99)
Patient 11-2 (1/100/100)
20.0
Time
Viral Load
Timepoint
Patient 15
Patient 15-14 (118/78/96)
Patient 15-19 (118/78/89)
Patient 15-15 (118/78/89)
Patient 15-23 (118/78/79)
Patient 15-17 (118/78/89)
Patient 15-21 (118/78/88)
Patient 15-24 (118/78/96)
Patient 15-16 (118/78/97)
Patient 15-13 (118/78/81)
Patient 15-20 (118/78/90)
Patient 15-18 (118/78/82)
Patient 15-22 (118/78/86)
Patient 15-11 (1/100/100)
Patient 15-1 (1/100/100)
Patient 15-12 (1/100/100)
Patient 15-10 (1/100/100)
Patient 15-2 (1/100/100)
Patient 15-3 (1/100/100)
Patient 15-9 (1/100/100)
Patient 15-8 (1/100/100)
Patient 15-5 (1/100/100)
Patient 15-6 (1/100/100)
30.0
Time
Viral Load
Timepoint
Patient 12
Patient 12-48 (226/57/76)
Patient 12-43 (226/57/70)
Patient 12-44 (226/57/81)
Patient 12-41 (226/57/61)
Patient 12-47 (226/57/62)
Patient 12-42 (226/57/63)
Patient 12-46 (226/57/69
Patient 12-39 (226/57/71)
Patient 12-45 (226/57/58)
Patient 12-37 (226/57/94)
Patient 12-40 (226/57/88)
Patient 12-38 (226/57/N/A)
Patient 12-25 (114/91/75)
Patient 12-31 (114/91/99)
Patient 12-28 (114/91/99)
Patient 12-32 (114/91/100)
Patient 12-33 (114/91/83)
Patient 12-35 (114/91/86)
Patient 12-29 (114/91/81)
Patient 12-17 (86/85/21)
Patient 12-20 (86/85/92)
Patient 12-14 (86/85/45)
Patient 12-15 (86/85/68)
Patient 12-19 (86/85/84)
Patient 12-16 (86/85/76)
Patient 12-21 (86/85/78)
Patient 12-24 (86/85/72)
Patient 12-13 (86/85/75)
Patient 12-18 (86/85/63)
Patient 12-10 (1/100/95)
Patient 12-7 (1/100/100)
Patient 12-8 (1/100/97)
Patient 12-12 (1/100/95)
Patient 12-6 (1/100/99)
Patient 12-11 (1/100/99)
Patient 12-4 (1/100/100)
Patient 12-3 (1/100/100)
Patient 12-5 (1/100/100)
Patient 12-2 (1/100/97)
Patient 12-1 (1/100/95)
Patient 12-9 (1/100/100)
40.0
Time
Viral Load
Timepoint
Patient 16
Patient 16-27 (353/41/48)
Patient 16-29 (353/41/58)
Patient 16-28 (353/41/56)
Patient 16-32 (353/41/56)
Patient 16-35 (353/41/95)
Patient 16-25 (353/41/56)
Patient 16-30 (353/41/51)
Patient 16-36 (353/41/49)
Patient 16-31 (353/41/56)
Patient 16-26 (353/41/52)
Patient 16-15 (87/98/99)
Patient 16-21 (87/98/98)
Patient 16-3 (1/96/98)
Patient 16-4 (1/96/96)
Patient 16-7 (1/96/99)
Patient 16-2 (1/96/97)
Patient 16-20 (87/98/99)
Patient 16-10 (1/96/98)
Patient 16-22 (87/98/99)
Patient 16-11 (1/96/99)
Patient 16-17 (87/98/100)
Patient 16-12 (1/96/96)
Patient 16-9 (1/96/95)
Patient 16-8 (1/96/98)
Patient 16-16 (87/98/99)
Patient 16-13 (87/98/99)
Patient 16-5 (1/96/99)
Patient 16-23 (87/98/99)
Patient 16-6 (1/96/100)
70.0
Time
Viral Load
Timepoint

Figure S2 C

Patient 18

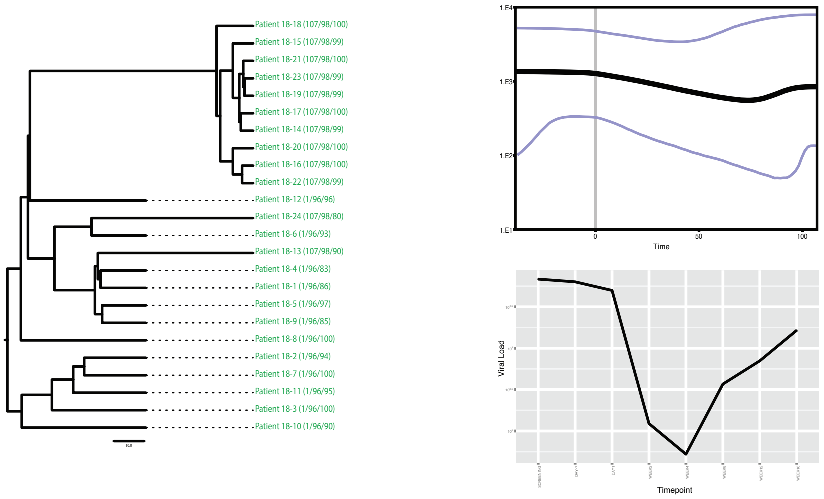

Patient 19

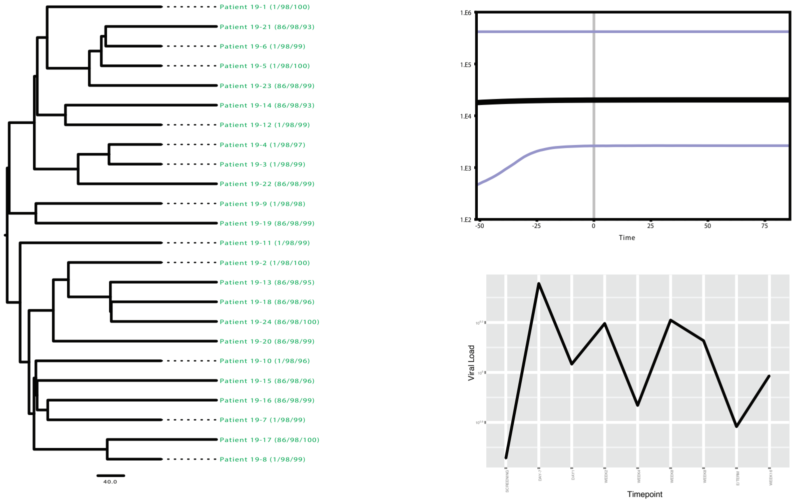

Patient 20

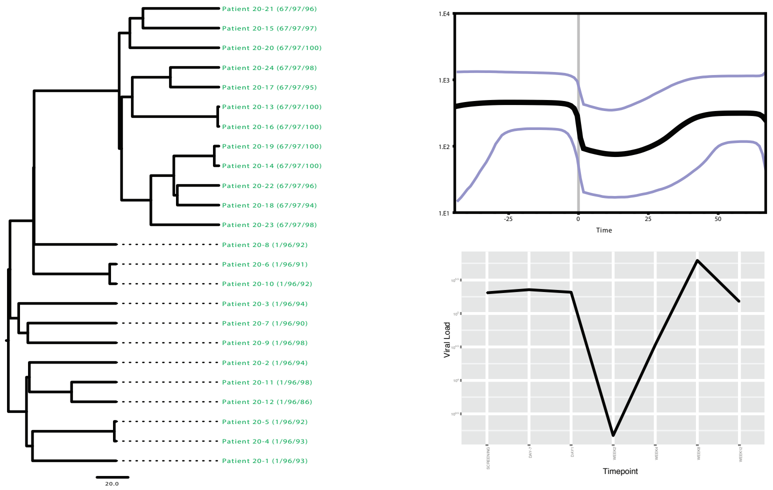

Figure S3. Covariation networks of virus from 18 patients. Sites in the V3 loop are labeled red in the network. There are seven patients with V3 loop involved in their networks (patient 1, 2, 5, 9, 15, 16 and 17). All sites in the network coevolve by both hydrophobicity and molecular weight. The size of the edges indicates the strength of covariation.

Figure S3

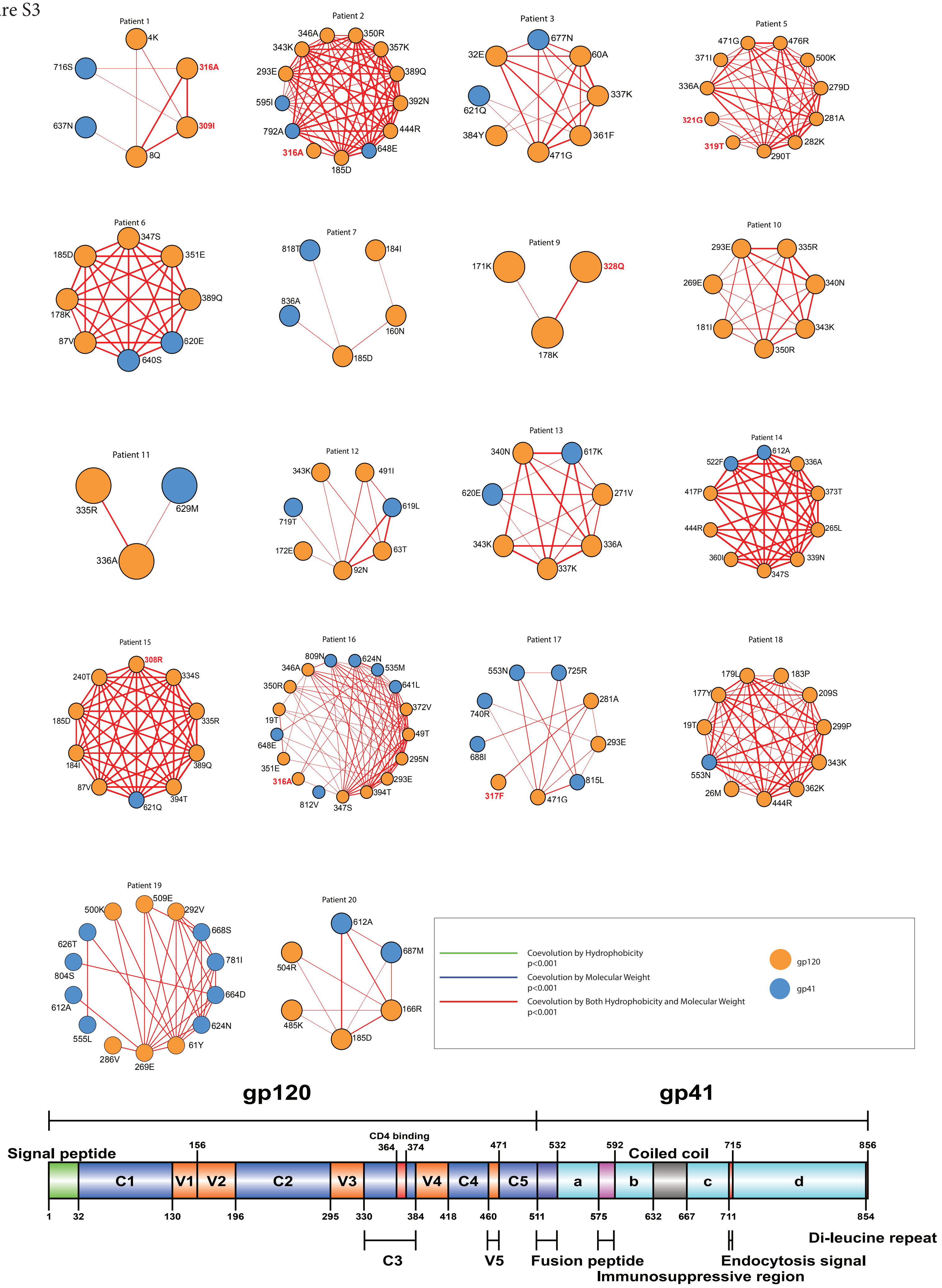

Patient 1
Patient 2
Patient 3
Patient 5
Patient 6
Patient 7
Patient 9
Patient 10
Patient 11
Patient 12
Patient 13
Patient 14
Patient 15
Patient 16
Patient 17
Patient 18
Patient 19
Patient 20
Coevolution by Hydrophobicity p<0.001
Coevolution by Molecular Weight p<0.001
Coevolution by Both Hydrophobicity and Molecular Weight p<0.001
gp120
gp41
Signal peptide
CD4 binding
Coiled coil
C1 V1 V2 C2 V3 V4 C4 C5 a b c d
C3
V5
Fusion peptide
Immunosuppressive region
Endocytosis signal
Di-leucine repeat

Figure S4. Covariation network from viruses when placebo-arm treatment started. The size of each circle indicates the relative number of interactions in the covariation networks. Information regarding positive selection, protein domain and glycosylation can be checked in supplementary table S5.

Figure S4

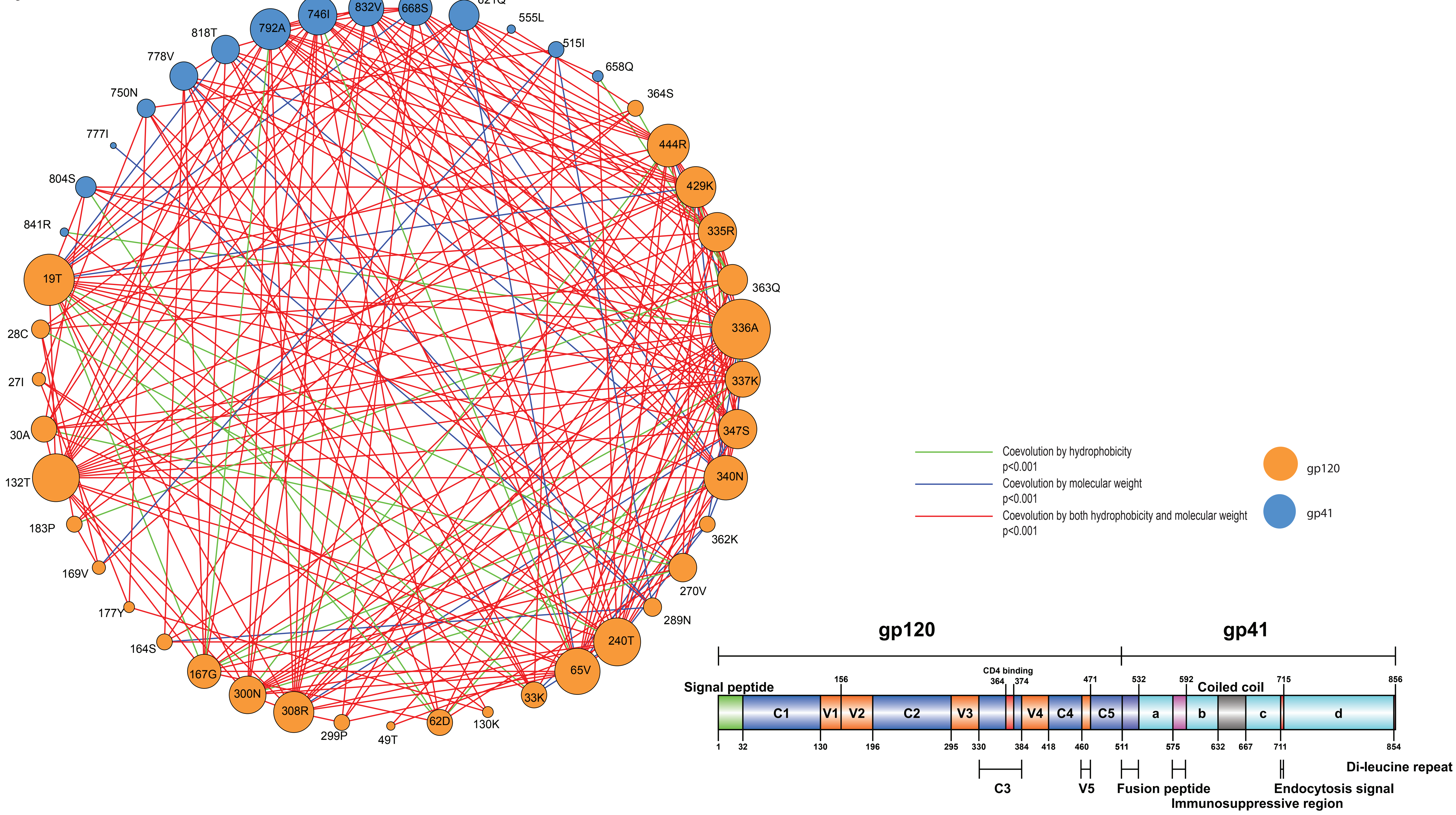

621Q
555L
515I
658Q
364S
444R
429K
335R
363Q
336A
337K
347S
340N
362K
270V
289N
240T
65V
33K
130K
62D
49T
299P
308R
300N
167G
164S
177Y
169V
183P
132T
30A
27I
28C
19T
841R
804S
777I
750N
778V
818T
792A
746I
832V
668S
Coevolution by hydrophobicity
p<0.001
Coevolution by molecular weight
p<0.001
Coevolution by both hydrophobicity and molecular weight
p<0.001
gp120
gp41
gp120
gp41
Signal peptide
CD4 binding
Coiled coil
156
364
374
471
532
592
715
856
C1
V1
V2
C2
V3
V4
C4
C5
a
b
c
d
1
32
130
196
295
330
384
418
460
511
575
632
667
711
854
C3
V5
Fusion peptide
Immunosuppressive region
Endocytosis signal
Di-leucine repeat

Figure S5. Covariation network from viruses after placebo-arm treatment. The size of each circle indicates the relative number of interactions in the covariation networks. Information regarding positive selection, protein domain and glycosylation can be checked in supplementary table S6.

Figure S5

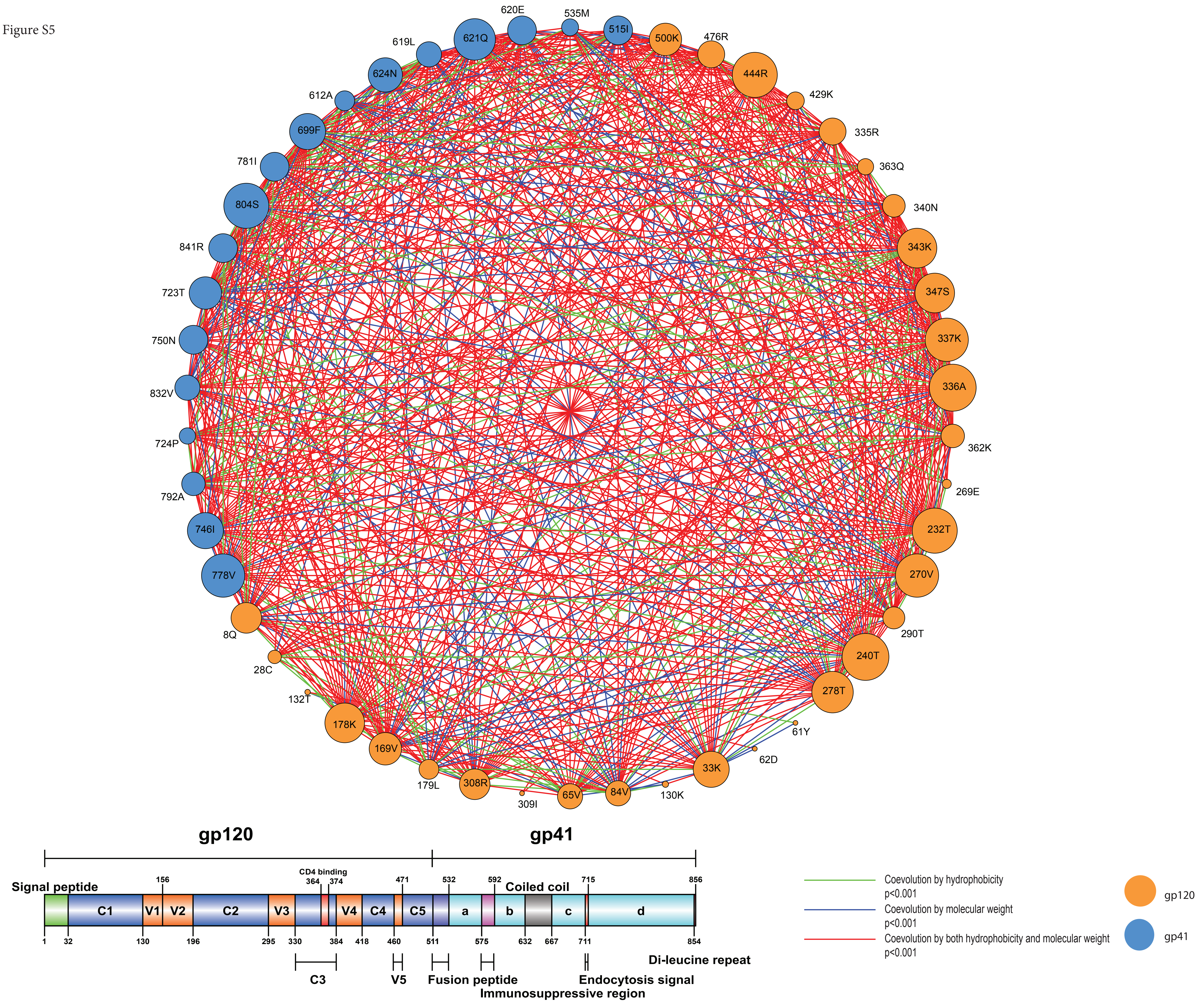
620E
535M
621Q
515I
500K
476R
619L
624N
444R
612A
429K
699F
335R
781I
363Q
804S
340N
841R
343K
723T
347S
750N
337K
832V
336A
724P
362K
792A
269E
746I
232T
778V
270V
8Q
290T
28C
240T
132T
278T
178K
61Y
169V
62D
179L
33K
308R
130K
309I
65V
84V
gp120
gp41
Signal peptide
156
CD4 binding
364
374
471
532
592
Coiled coil
715
856
C1
V1
V2
C2
V3
V4
C4
C5
a
b
c
d
1
32
130
196
295
330
384
418
460
511
575
632
667
711
854
C3
V5
Fusion peptide
Immunosuppressive region
Endocytosis signal
Di-leucine repeat
Coevolution by hydrophobicity p<0.001
Coevolution by molecular weight p<0.001
Coevolution by both hydrophobicity and molecular weight p<0.001
gp120
gp41

Figure S6. A network of covarying sites under positive selection unique to maraviroc-resistant virus. (A) The size of each circle indicates the relative number of interactions in the covariation network. All sites are positively selected ($p<0.05$) and they are unique to the resistant virus. Covarying sites are connected by lines with three different colours indicating their nature of covariation. Black line indicates the two sites covary by BLOSUM62 scores. Green line indicates the two sites covary by hydrophobicity. Blue line indicates the two sites covary by molecular weight. Finally, red line indicates the two sites covary by both hydrophobicity and molecular weight. The location of each site can be checked in (B). All covarying sites are mapped into a hypothetical structural complex CD4-GP120-CCR5 (Figure S7).

Figure S6

A

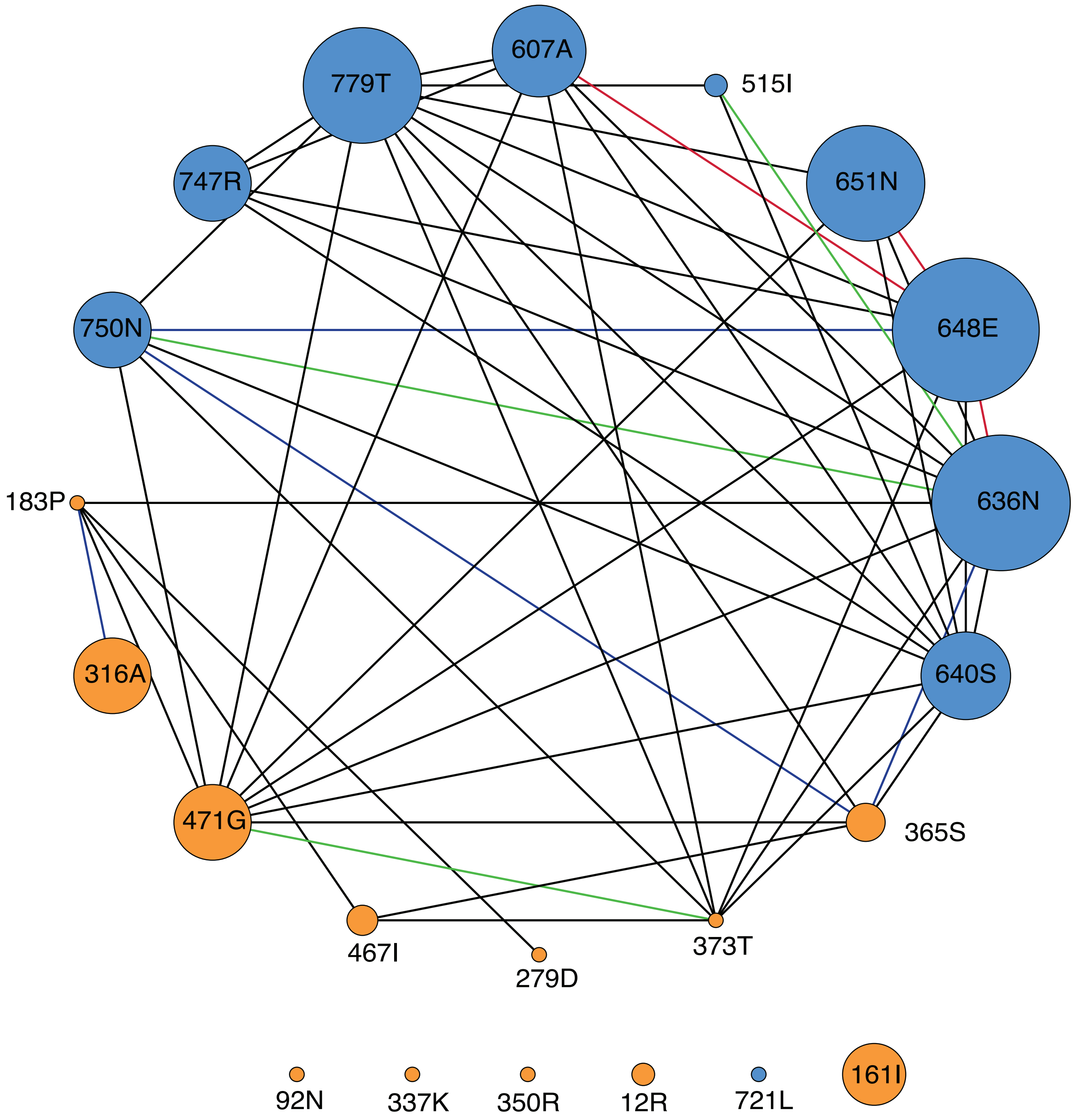
607A
779T
515I
651N
747R
648E
750N
636N
183P
316A
640S
471G
365S
467I
373T
279D
92N
337K
350R
12R
721L
161I

B

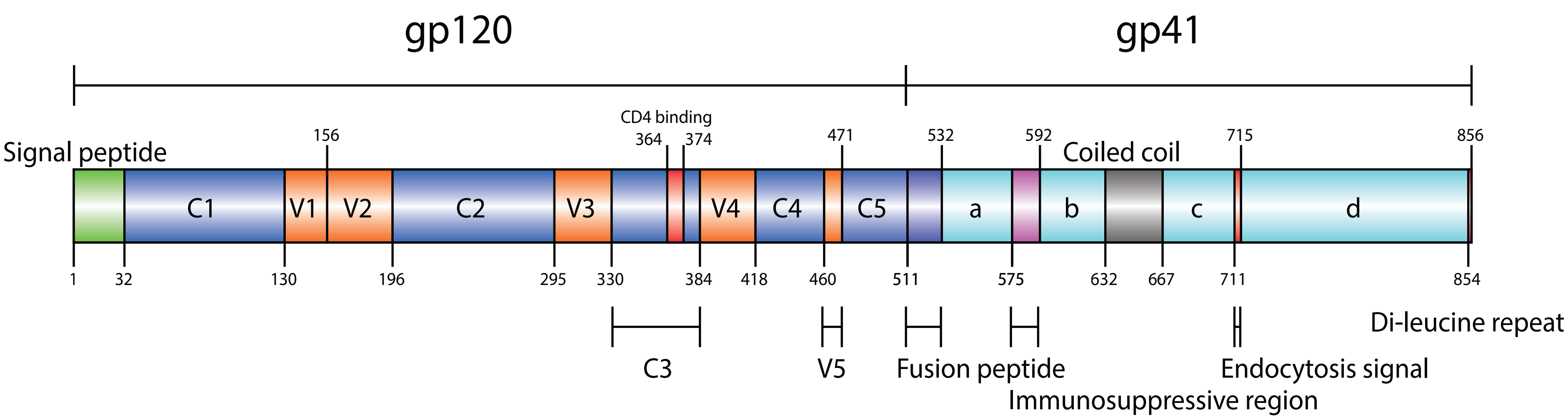
gp120
gp41
Signal peptide
CD4 binding
156
364
374
471
532
592
Coiled coil
715
856
C1
V1
V2
C2
V3
V4
C4
C5
a
b
c
d
1
32
130
196
295
330
384
418
460
511
575
632
667
711
854
Di-leucine repeat
C3
V5
Fusion peptide
Endocytosis signal
Immunosuppressive region

Figure S7. Coevolving sites under positive selection and unique to resistant virus mapped to a hypothetical CD4-GP120-CCR5 structural complex. The complex is shown in A with CD4, GP120, CCR5 and with two N-linked glycans modelled in Table S8 and Figure S6. All coevolving sites are labelled with HXB2 reference amino acids and shown in coloured spheres. Two key interactions, gp120-CD4 and V3-ECL2 (the second extracellular loop of CCR5, coloured red in A and C), are zoomed in B and C, respectively. CD4-GP120-CCR5 complex is based on PDB 2QAD (1) and a CCR5 PDB structure from (2). The two N-glycans (mannose 5) are modelled by GLYCAM (3) and only mannose 5 is modelled (4). The figure was prepared with PyMOL 1.7.5.

1. **Huang CC, Lam SN, Acharya P, Tang M, Xiang SH, Hussan SS, Stanfield RL, Robinson J, Sodroski J, Wilson IA, Wyatt R, Bewley CA, Kwong PD.** 2007. Structures of the CCR5 N terminus and of a tyrosine-sulfated antibody with HIV-1 gp120 and CD4. Science **317:**1930-1934.

2. **Garcia-Perez J, Rueda P, Alcami J, Rognan D, Arenzana-Seisdedos F, Lagane B, Kellenberger E.** 2011. Allosteric model of maraviroc binding to CC chemokine receptor 5 (CCR5). J Biol Chem **286:**33409-33421.

3. **Kirschner KN, Yongye AB, Tschampel SM, Gonzalez-Outeirino J, Daniels CR, Foley BL, Woods RJ.** 2008. GLYCAM06: a generalizable biomolecular force field. Carbohydrates. J Comput Chem **29:**622-655.

4. **Pancera M, Zhou T, Druz A, Georgiev IS, Soto C, Gorman J, Huang J, Acharya P, Chuang GY, Ofek G, Stewart-Jones GB, Stuckey J, Bailer RT, Joyce MG, Louder MK, Tumba N, Yang Y, Zhang B, Cohen MS, Haynes BF, Mascola JR, Morris L, Munro JB, Blanchard SC, Mothes W, Connors M, Kwong PD.** 2014. Structure and immune recognition of trimeric pre-fusion HIV-1 Env. Nature **514:**455-461.

Figure S7

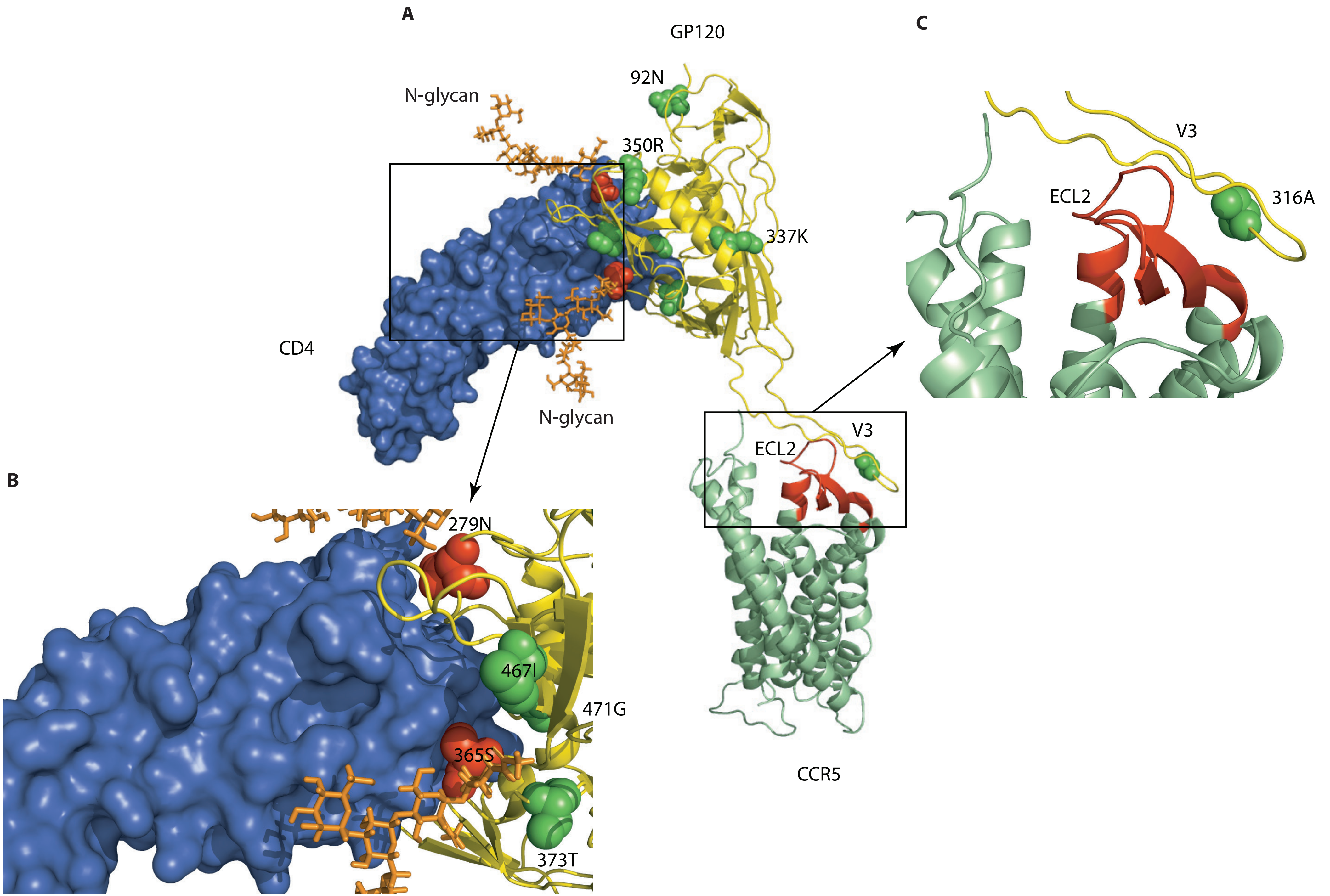
A
GP120
92N
N-glycan
350R
337K
CD4
N-glycan
V3
ECL2
CCR5
C
V3
ECL2
316A
B
279N
467I
471G
365S
373T
